# Supplementary material for: Insertional Inactivation of Prevotella intermedia OxyR Results in Reduced Survival with Oxidative Stress and in the Presence of Host Cells
Source: Microorganisms. 2021 Mar 7;9(3):551. doi: 10.3390/microorganisms9030551 (PMC7999485; doi:10.3390/microorganisms9030551)
Supplement: Supplementary file 1 [file microorganisms-09-00551-s001.pdf]

Supplementary Material

Supplementary Tables:

Supplementary Table S1. Table 1. List of bacterial strains used in this study

Supplementary Table S2. List of plasmids used in this study

Supplementary Table 3. List of primers used for mutagenesis of *P. intermedia*

Supplementary Table 4. List of primers used for qRT-PCR analysis in *P. intermedia*

Supplementary Table 5. List of the most highly upregulated genes in *P. intermedia* OxyR mutant

Supplementary Table 6. List of the most highly downregulated genes in *P. intermedia* OxyR mutant

Supplementary Table 7. List of the most highly upregulated genes in *P. intermedia* grown in iron-deplete conditions

Supplementary Table 8. List of the most highly downregulated genes in *P. intermedia* grown in iron-deplete conditions

Supplementary Figures:

Supplementary Figure 1. Comparison of the genomic loci encoding OxyR in *Prevotella* species.

Supplementary Figure 2. Distribution of SOD and glutathione peroxidase genes within the genus *Prevotella*.

Supplementary Table S1. Bacterial strains

Strain	Description	Source or reference
P. intermedia		
V3147	Wild type OMA14 isolated from the periodontal pocket of a Japanese patient with periodontitis	(1)
V3203	OMA14 PIOMA14_I_0073(oxyR*)*::*ermF*	This study
E. coli		
XL-1 Blue	Host strain for cloning	Stratagene
S17-1	RP-4-2-Tc::Mu *aph*::Tn7 *recA*, Smr	(2)

Supplementary Table S2. Plasmids

Plasmid	Relevant property	Source or reference
pUC118		Takara
pBSSK		
pNDR-Dual		Clonetech
pTCB	Ap^r Tc^r, *E. coli-Bacteroides* shuttle vector plasmid	(3)
pKD954	Contains the *Porpyromonas gulae* catalase promoter in pBSSK	(4)
pKD718	*ermF* inserted into the BamHI site of pGEM Easy vector	(5)
pUC118-sacB	*sacB* inserted into the HincII site of pUC118	This study
pUC118-Pcat	*Porpyromonas gulae* catalase promoter (*Pcat*) inserted into the HincII site of pUC118	This study
pUC118-Pcat-sacB	*Pcat-sacB* inserted into the HincII site of pUC118	This study
pDNK0001	Contains upstream (913 bp) and downstream (822 bp) regions of PIOMA14_I_0073 (*oxyR*) with HincII site in pBSSK	This study
pDNK0002	*ermF* inserted into the BglII site and Pcat-sacB inserted into the BamHI site of pND0001	This study
pDNK0003	NotI-XhoI fragment of pND0002 inserted into the NotI-XhoI site of pTCB	This study

Supplementary Table S3. Primers

Target region	Primer name	Sequence
ermF	ermF-5’	GGATCCcccgatagcttcc
	ermF-3’	GGATCCcctacgaaggatgaaattt
sacB	sacB-N-BglII	AGATCTatgaacatcaaaaagtttgcaaaacaag
	sacB-C-BamHI	GGATCCttatttgttaactgttaattgtccttg
Porpyromonas gulae catalase promoter	p6-34-F-BglII	AGATCTttcgtcgtcaatcagcatcccag
	p6-34-R-BamHI	GGATCCtgttttgtctcttatttaagtta
PIOMA14_I_0073 (*oxyR*)	oxyR-dw-R-BamHI	GAATTCgttatcagcaccaacagg
	oxyR-dw-F-BglII	gttgAGATCTagtagccgagagccttta
	oxyR-up-R-BglII	tactAGATCTcaacctttagaggaaatgct
	oxyR-up-F-EcoRI	GGATCCggcttacttcatactacgc
	86467F	ggttgtacttagccatacggtc
	90779R	ctaaAGATCTcctattgagaagacacaggac

Capital letters indicate the added restriction enzyme recognition site. up: upstream, dw; downstream, F; forward primer, R; reverse primer.

Supplementary Table S4. Primers used for qRT-PCR analysis.

**Primer Target**	**Sequence (5'-3')**
AhpC-F	CCTACGAGGTCGATGCTTGG
AhpC-R	GGTTAAGGCTGCACAGTTCG
AhpF-F	AGTCTGCGCCAGTTTGCACC
AhpF-R	AACACAGCTTGCTGCCGACC
OxyR-F	TGTCGCCGTGTTTCAGTGCC
OxyR-R	TTGCCCACCATCGCCCCTTA
Ferritin-F	ACGGTCCTTCTCCTCGTCGG
Ferritin-R	CGTGGCGGTGTGCCTACAAT
cydA-F	TGGCACGCGGTAAGCAAGCC
cydA-R	GGAGGCACGGTTTCGTCGGC
Dps-F	ACCTCATTGCACAAGAGCGTGC
Dps-R	ATGGTGGTGTTCAGCGAAAGCG

Supplemental references

1. Naito M, Ogura Y, Itoh T, Shoji M, Okamoto M, Hayashi T, Nakayama K. 2016. The complete genome sequencing of *Prevotella intermedia* strain OMA14 and a subsequent fine-scale, intra-species genomic comparison reveal an unusual amplification of conjugative and mobile transposons and identify a novel *Prevotella*-lineage-specific repeat. DNA Res 23:11-19.
2. Simon R, Priefer U, Pühler A. 1983. A broad host range mobilization system for *in vivo* genetic engineering: transposon mutagenesis in Gram negative bacteria. Nat Technol 1:784-791.
3. Nagano K, Murakami Y, Nishikawa K, Sakakibara J, Shimozato K, Yoshimura F. 2007. Characterization of RagA and RagB in *Porphyromonas gingivalis*: study using gene-deletion mutants. J Med Microbiol 56:1536-1548.
4. Sato K, Naito M, Yukitake H, Hirakawa H, Shoji M, McBride MJ, Rhodes RG, Nakayama K. 2009. A protein secretion system linked to bacteroidete gliding motility and pathogenesis. Proc Natl Acad Sci U S A 107:276-281.
5. Yoshimura M, Ohara N, Kondo Y, Shoji M, Okano S, Nakano Y, Abiko Y, Nakayama K. 2008. Proteome analysis of *Porphyromonas gingivalis* cells placed in a subcutaneous chamber of mice. Oral Microbiol Immunol 23:413-418.

Supplementary Table S5. Genes downregulated in *P. intermedia* OMA14 OxyR mutant (2 ≥ fold, P≤0.1)

locus tag	old locus tag	gene name	[1]Max group mean	[2]Log_2 fold change	[2]Fold change	[3]P-value	[4]FDR p-value	product (Genome (CDS))
PIOMA14_RS00365	PIOMA14_I_0073	oxyR	118.26	-9.99	-1016.60	1.93E-02	2.86E-01	hydrogen peroxide-inducible genes activator
PIOMA14_RS00355	PIOMA14_I_0070	ahpC	1264.78	-8.56	-377.65	6.66E-16	8.54E-13	peroxiredoxin
PIOMA14_RS00350	PIOMA14_I_0069	ahpF	2036.73	-7.26	-153.14	0.00E+00	0.00E+00	alkyl hydroperoxide reductase subunit F
PIOMA14_RS09985	PIOMA14_I_1962		495.03	-4.19	-18.30	2.68E-13	2.29E-10	DNA starvation/stationary phase protection protein
PIOMA14_RS12670	PIOMA14_II_0482		56.43	-3.85	-14.42	6.61E-04	3.68E-02	DUF3408 domain-containing protein, conjugal transfer protein TraA
PIOMA14_RS11405	PIOMA14_II_0222	queG	244.31	-2.94	-7.67	3.06E-08	1.12E-05	tRNA epoxyqueuosine(34) reductase QueG, polysaccharide deacetylase family protein
PIOMA14_RS11595	PIOMA14_II_0270		56.86	-2.85	-7.20	1.12E-03	5.40E-02	conjugal transfer protein TraA, DUF3408 domain-containing protein
PIOMA14_RS07945	PIOMA14_I_1570		27.77	-2.84	-7.18	2.81E-03	1.00E-01	hypothetical protein
PIOMA14_RS04640	PIOMA14_I_0913		31.07	-2.78	-6.86	1.03E-02	2.11E-01	hypothetical protein
PIOMA14_RS02545	PIOMA14_I_0510		32.93	-2.75	-6.73	2.06E-04	1.67E-02	GNAT family N-acetyltransferase, glycosyltransferase family 2 protein, hypothetical protein
PIOMA14_RS12690	PIOMA14_II_0488		44.89	-2.64	-6.22	2.22E-05	3.56E-03	YWFCY domain-containing protein
PIOMA14_RS04625	PIOMA14_I_0910		52.85	-2.60	-6.04	7.72E-03	1.78E-01	ABC transporter ATP-binding protein
PIOMA14_RS11575	PIOMA14_II_0264		39.95	-2.56	-5.89	1.42E-05	2.43E-03	YWFCY domain-containing protein
PIOMA14_RS01405	PIOMA14_I_0285		15.29	-2.49	-5.60	3.30E-03	1.08E-01	peptidase domain-containing ABC transporter
PIOMA14_RS07930	PIOMA14_I_1567		52.18	-2.42	-5.36	9.60E-03	2.03E-01	ABC transporter ATP-binding protein
PIOMA14_RS07695	PIOMA14_I_1514		54.56	-2.42	-5.35	8.94E-03	1.97E-01	ABC transporter ATP-binding protein
PIOMA14_RS11555	PIOMA14_II_0258		43.36	-2.39	-5.24	1.17E-02	2.30E-01	hypothetical protein
PIOMA14_RS11415	PIOMA14_II_0224		210.28	-2.37	-5.16	1.13E-09	5.79E-07	DUF2723 domain-containing protein
PIOMA14_RS12695	PIOMA14_II_0490		1308.58	-2.37	-5.16	9.26E-05	9.13E-03	toxin PIN
PIOMA14_RS14600	PIOMA14_II_0260		107.72	-2.35	-5.10	1.45E-02	2.60E-01	hypothetical protein
PIOMA14_RS03940	PIOMA14_I_0776		260.05	-2.33	-5.02	3.86E-05	5.82E-03	DNA-3-methyladenine glycosylase I
PIOMA14_RS09210	PIOMA14_I_1816		18.15	-2.31	-4.95	3.48E-02	3.69E-01	DedA family protein
PIOMA14_RS03545	PIOMA14_I_0697	carA	79.36	-2.31	-4.94	8.28E-05	8.51E-03	glutamine-hydrolyzing carbamoyl-phosphate synthase small subunit, carbamoyl-phosphate synthase (glutamine-hydrolyzing) large subunit
PIOMA14_RS03345	PIOMA14_I_0663		7.73	-2.17	-4.51	5.54E-02	4.37E-01	DUF4359 domain-containing protein
PIOMA14_RS11570	PIOMA14_II_0262		1140.96	-2.17	-4.50	2.41E-04	1.87E-02	toxin PIN
PIOMA14_RS11410	PIOMA14_II_0223		116.66	-2.16	-4.46	4.93E-05	6.32E-03	tRNA epoxyqueuosine(34) reductase QueG, polysaccharide deacetylase family protein
PIOMA14_RS07680	PIOMA14_I_1511		31.19	-2.14	-4.41	4.28E-02	4.01E-01	hypothetical protein
PIOMA14_RS03835	PIOMA14_I_0754		525.21	-2.06	-4.18	8.30E-05	8.51E-03	chitobiase/beta-hexosaminidase C-terminal domain-containing protein
PIOMA14_RS08830	PIOMA14_I_1746		135.26	-2.06	-4.18	2.78E-04	2.03E-02	hypothetical protein
PIOMA14_RS04075	PIOMA14_I_0800		1256.15	-2.05	-4.14	2.09E-04	1.67E-02	DEAD/DEAH box helicase
PIOMA14_RS12700	PIOMA14_II_0491		512.63	-2.05	-4.13	3.69E-07	1.05E-04	peptidase M26
PIOMA14_RS09975	PIOMA14_I_1960	queA	44.22	-2.00	-4.00	3.13E-03	1.04E-01	tRNA pseudouridine(55) synthase TruB, tRNA preQ1(34) S-adenosylmethionine ribosyltransferase-isomerase QueA
PIOMA14_RS02540	PIOMA14_I_0509		119.35	-1.99	-3.98	1.05E-04	9.58E-03	GNAT family N-acetyltransferase, glycosyltransferase family 2 protein
PIOMA14_RS01970	PIOMA14_I_0390		28.80	-1.99	-3.98	1.40E-02	2.58E-01	nucleotidyltransferase family protein
PIOMA14_RS07685	PIOMA14_I_1512		12.54	-1.99	-3.97	8.04E-02	5.09E-01	outer membrane lipoprotein-sorting protein
PIOMA14_RS01400	PIOMA14_I_0284		88.68	-1.92	-3.78	5.67E-05	6.92E-03	outer membrane beta-barrel protein
PIOMA14_RS11565	PIOMA14_II_0261		491.49	-1.92	-3.78	1.43E-06	3.34E-04	peptidase M26
PIOMA14_RS03035	PIOMA14_I_0605		505.79	-1.92	-3.78	9.85E-02	5.34E-01	TetR/AcrR family transcriptional regulator
PIOMA14_RS00400	PIOMA14_I_0080		20.94	-1.91	-3.76	8.94E-02	5.29E-01	lipocalin-like domain-containing protein
PIOMA14_RS09000	PIOMA14_I_1779		3.49	-1.91	-3.75	9.28E-02	5.33E-01	GH32 C-terminal domain-containing protein
PIOMA14_RS08765	PIOMA14_I_1735		12.01	-1.89	-3.71	3.69E-02	3.83E-01	redoxin domain-containing protein
PIOMA14_RS02355	PIOMA14_I_0471		54.41	-1.89	-3.70	5.76E-03	1.43E-01	AraC family transcriptional regulator
PIOMA14_RS02080	PIOMA14_I_0412		103.23	-1.88	-3.69	2.82E-02	3.51E-01	hypothetical protein
PIOMA14_RS03540	PIOMA14_I_0696		571.89	-1.87	-3.66	2.01E-07	6.45E-05	amidophosphoribosyltransferase
PIOMA14_RS04885	PIOMA14_I_0961		36.77	-1.86	-3.62	3.08E-02	3.66E-01	hypothetical protein
PIOMA14_RS02375	PIOMA14_I_0476		189.54	-1.84	-3.58	6.01E-04	3.50E-02	hypothetical protein
PIOMA14_RS07855	PIOMA14_I_1548		1002.50	-1.80	-3.49	4.47E-06	8.18E-04	Omp28-related outer membrane protein
PIOMA14_RS06110	PIOMA14_I_1198		58.59	-1.80	-3.48	1.27E-02	2.45E-01	ADP-ribosylglycohydrolase family protein
PIOMA14_RS03275	PIOMA14_I_0653		122.96	-1.80	-3.48	8.60E-04	4.59E-02	HAD family hydrolase
PIOMA14_RS12705	PIOMA14_II_0493		890.54	-1.80	-3.47	1.04E-03	5.25E-02	hypothetical protein
PIOMA14_RS11560	PIOMA14_II_0259		870.89	-1.77	-3.40	1.22E-03	5.81E-02	hypothetical protein
PIOMA14_RS07860	PIOMA14_I_1549		98.49	-1.76	-3.39	4.57E-04	2.85E-02	C10 family peptidase
PIOMA14_RS03775	PIOMA14_I_0743		730.15	-1.75	-3.37	6.66E-05	7.63E-03	hypothetical protein, phosphotransferase
PIOMA14_RS06105	PIOMA14_I_1197		224.33	-1.72	-3.28	6.51E-02	4.65E-01	T9SS type A sorting domain-containing protein
PIOMA14_RS00550	PIOMA14_I_0110		23.93	-1.71	-3.26	1.62E-02	2.62E-01	MATE family efflux transporter
PIOMA14_RS09205	PIOMA14_I_1815		85.59	-1.69	-3.23	3.12E-03	1.04E-01	MATE family efflux transporter
PIOMA14_RS08820	PIOMA14_I_1744		128.98	-1.67	-3.17	7.24E-04	3.95E-02	RagB/SusD family nutrient uptake outer membrane protein
PIOMA14_RS07700	PIOMA14_I_1515		71.37	-1.66	-3.17	4.54E-02	4.04E-01	ABC transporter ATP-binding protein
PIOMA14_RS03935	PIOMA14_I_0775		358.46	-1.66	-3.17	4.46E-05	6.32E-03	NAD(P)/FAD-dependent oxidoreductase
PIOMA14_RS02605	PIOMA14_I_0523		199.55	-1.64	-3.13	2.41E-03	8.94E-02	hypothetical protein
PIOMA14_RS04620	PIOMA14_I_0909		65.59	-1.64	-3.12	9.85E-02	5.34E-01	ABC transporter ATP-binding protein
PIOMA14_RS01690	PIOMA14_I_0341		48.50	-1.64	-3.11	6.28E-02	4.65E-01	tellurium resistance protein TerC, transcriptional regulator
PIOMA14_RS02765	PIOMA14_I_0553		149.99	-1.63	-3.09	2.61E-03	9.43E-02	nucleoside deaminase
PIOMA14_RS01265		lpxA_1	53.12	-1.62	-3.08	5.22E-03	1.38E-01	acyl-ACP--UDP-N-acetylglucosamine O-acyltransferase
PIOMA14_RS03770	PIOMA14_I_0742		825.77	-1.60	-3.03	3.77E-04	2.54E-02	hypothetical protein, phosphotransferase
PIOMA14_RS11850	PIOMA14_II_0320		38.96	-1.60	-3.03	8.71E-02	5.28E-01	DUF4296 domain-containing protein, hypothetical protein
PIOMA14_RS03790	PIOMA14_I_0746	mscL	194.63	-1.59	-3.01	3.82E-03	1.22E-01	large-conductance mechanosensitive channel protein MscL
PIOMA14_RS01260	PIOMA14_I_0255	miaA_1	150.15	-1.58	-2.99	3.54E-03	1.15E-01	tRNA (adenosine(37)-N6)-dimethylallyltransferase MiaA

PIOMA14_RS02730	PIOMA14_I_0546		65.85	-1.56	-2.95	2.43E-02	3.26E-01	DUF4293 domain-containing protein, DNA-directed RNA polymerase subunit omega
PIOMA14_RS14625	PIOMA14_II_0492		117.51	-1.55	-2.93	5.87E-02	4.42E-01	hypothetical protein
PIOMA14_RS03535	PIOMA14_I_0695	glmS	332.92	-1.53	-2.88	6.84E-05	7.63E-03	glutamine-fructose-6-phosphate transaminase (isomerizing)
PIOMA14_RS01395	PIOMA14_I_0283		126.58	-1.52	-2.88	7.69E-03	1.78E-01	radical SAM protein
PIOMA14_RS02370	PIOMA14_I_0475		173.81	-1.51	-2.85	6.40E-04	3.65E-02	peptidase M26
PIOMA14_RS08120	PIOMA14_I_1606		95.89	-1.51	-2.85	4.35E-03	1.34E-01	nucleoside recognition domain-containing protein
PIOMA14_RS03550	PIOMA14_I_0698	carB	113.89	-1.51	-2.84	1.57E-02	2.60E-01	glutamine-hydrolyzing carbamoyl-phosphate synthase small subunit, carbamoyl-phosphate synthase (glutamine-hydrolyzing) large subunit
PIOMA14_RS01920	PIOMA14_I_0380		63.77	-1.48	-2.79	5.64E-02	4.37E-01	HU family DNA-binding protein
PIOMA14_RS06100	PIOMA14_I_1196		306.52	-1.48	-2.79	9.32E-02	5.34E-01	HmuY family protein
PIOMA14_RS10165	PIOMA14_I_1994		150.86	-1.46	-2.75	5.80E-03	1.43E-01	adenosylcobalamin-dependent ribonucleoside-diphosphate reductase
PIOMA14_RS12710	PIOMA14_II_0494		48.92	-1.44	-2.72	7.10E-02	4.83E-01	hypothetical protein
PIOMA14_RS03960	PIOMA14_I_0779		582.27	-1.44	-2.71	3.86E-03	1.22E-01	SpoIID/LytB domain-containing protein
PIOMA14_RS08825	PIOMA14_I_1745		356.79	-1.43	-2.70	1.10E-02	2.23E-01	SusF/SusE family outer membrane protein
PIOMA14_RS01325	PIOMA14_I_0268		487.76	-1.43	-2.70	1.38E-03	6.03E-02	UvrD-helicase domain-containing protein
PIOMA14_RS03530	PIOMA14_I_0694		165.01	-1.42	-2.68	6.77E-03	1.59E-01	polysaccharide biosynthesis/export family protein
PIOMA14_RS04690	PIOMA14_I_0925	menB	22.69	-1.40	-2.64	6.16E-02	4.59E-01	1,4-dihydroxy-2-naphthoyl-CoA synthase
PIOMA14_RS03705	PIOMA14_I_0731		222.58	-1.38	-2.60	2.77E-02	3.50E-01	dCMP deaminase family protein, S41 family peptidase
PIOMA14_RS03070	PIOMA14_I_0612	gcvT	79.06	-1.37	-2.58	3.67E-02	3.83E-01	glycine cleavage system aminomethyltransferase GcvT
PIOMA14_RS03930	PIOMA14_I_0774		250.39	-1.37	-2.58	1.01E-02	2.11E-01	YggS family pyridoxal phosphate-dependent enzyme
PIOMA14_RS10405	PIOMA14_II_0046		250.76	-1.36	-2.56	4.95E-03	1.38E-01	ATP-dependent Clp protease ATP-binding subunit, hypothetical protein
PIOMA14_RS08805	PIOMA14_I_1741		32.21	-1.36	-2.56	1.05E-02	2.14E-01	SLC45 family MFS transporter
PIOMA14_RS03055	PIOMA14_I_0609	gcvPB	198.30	-1.35	-2.55	5.65E-02	4.37E-01	aminomethyl-transferring glycine dehydrogenase subunit GcvPB
PIOMA14_RS07455	PIOMA14_I_1467	pnp	1060.58	-1.33	-2.52	1.32E-02	2.53E-01	polyribonucleotide nucleotidyltransferase
PIOMA14_RS04385	PIOMA14_I_0859		18.98	-1.32	-2.50	6.75E-02	4.73E-01	calcium/sodium antiporter
PIOMA14_RS04890	PIOMA14_I_0962		32.52	-1.31	-2.49	8.12E-03	1.84E-01	glycosyl hydrolase family 25
PIOMA14_RS10070	PIOMA14_I_1975	xth	82.67	-1.28	-2.44	5.47E-03	1.39E-01	exodeoxyribonuclease III
PIOMA14_RS00910	PIOMA14_I_0185		358.00	-1.28	-2.43	4.86E-03	1.38E-01	F0F1 ATP synthase subunit delta
PIOMA14_RS03695	PIOMA14_I_0729		270.93	-1.26	-2.40	5.91E-03	1.43E-01	Crp/Fnr family transcriptional regulator
PIOMA14_RS03855	PIOMA14_I_0759		48.19	-1.26	-2.40	3.57E-02	3.76E-01	Cof-type HAD-IIB family hydrolase
PIOMA14_RS08920	PIOMA14_I_1762		231.64	-1.26	-2.39	5.89E-03	1.43E-01	LicD family protein
PIOMA14_RS12410	PIOMA14_II_0430		120.22	-1.26	-2.39	2.97E-02	3.60E-01	hypothetical protein
PIOMA14_RS04020	PIOMA14_I_0790		595.26	-1.25	-2.38	4.44E-02	4.01E-01	YigZ family protein
PIOMA14_RS02070	PIOMA14_I_0410		20.45	-1.25	-2.37	8.39E-02	5.18E-01	ATP-grasp domain-containing protein
PIOMA14_RS09185	PIOMA14_I_1811		116.67	-1.24	-2.36	2.64E-02	3.40E-01	S41 family peptidase, DUF3316 domain-containing protein
PIOMA14_RS03785	PIOMA14_I_0745	guaA	289.25	-1.24	-2.36	1.58E-02	2.60E-01	glutamine-hydrolyzing GMP synthase
PIOMA14_RS06945	PIOMA14_I_1264		52.27	-1.23	-2.35	1.01E-01	5.35E-01	transporter, SPFH domain-containing protein
PIOMA14_RS10905	PIOMA14_II_0132		128.70	-1.23	-2.34	4.65E-03	1.37E-01	S-adenosylmethionine:tRNA ribosyltransferase-isomerase
PIOMA14_RS05455	PIOMA14_I_1076	rpsH	59.80	-1.23	-2.34	9.06E-02	5.30E-01	30S ribosomal protein S8
PIOMA14_RS13700	PIOMA14_II_0711		62.90	-1.22	-2.34	2.02E-02	2.90E-01	deoxynucleoside kinase
PIOMA14_RS03865	PIOMA14_I_0761		253.91	-1.22	-2.33	1.55E-02	2.60E-01	patatin-like phospholipase family protein
PIOMA14_RS00280	PIOMA14_I_0058		21.91	-1.22	-2.33	9.73E-02	5.34E-01	hypothetical protein
PIOMA14_RS03060	PIOMA14_I_0610	gcvPA	109.76	-1.22	-2.32	2.12E-02	2.99E-01	aminomethyl-transferring glycine dehydrogenase subunit GcvPA
PIOMA14_RS03850	PIOMA14_I_0757		720.67	-1.22	-2.32	2.60E-02	3.36E-01	C10 family peptidase
PIOMA14_RS03690	PIOMA14_I_0728		108.14	-1.21	-2.31	2.92E-02	3.57E-01	FAD:protein FMN transferase
PIOMA14_RS03490	PIOMA14_I_0687		20.31	-1.19	-2.28	1.00E-01	5.35E-01	DMT family transporter
PIOMA14_RS00540	PIOMA14_I_0108		301.92	-1.18	-2.27	2.82E-02	3.51E-01	metallophosphoesterase family protein
PIOMA14_RS10505	PIOMA14_II_0066	nqrC	59.90	-1.18	-2.26	4.26E-02	4.01E-01	NADH:ubiquinone reductase (Na(+)-transporting) subunit F
PIOMA14_RS04380	PIOMA14_I_0858		315.45	-1.18	-2.26	1.89E-02	2.86E-01	cation:proton antiporter
PIOMA14_RS10490	PIOMA14_II_0063		278.69	-1.18	-2.26	1.03E-02	2.11E-01	NADH:ubiquinone reductase (Na(+)-transporting) subunit F
PIOMA14_RS04925	PIOMA14_I_0969	pgeF	296.64	-1.17	-2.25	6.37E-02	4.65E-01	GTPase ObgE, peptidoglycan editing factor PgeF
PIOMA14_RS05460	PIOMA14_I_1077	rpsN	668.70	-1.17	-2.25	5.44E-02	4.37E-01	30S ribosomal protein S14
PIOMA14_RS02610	PIOMA14_I_0524	def	267.36	-1.16	-2.23	3.24E-02	3.69E-01	peptide deformylase
PIOMA14_RS13490	PIOMA14_II_0667		34.61	-1.16	-2.23	9.93E-02	5.34E-01	cell envelope biogenesis protein LolA
PIOMA14_RS09750	PIOMA14_I_1920		72.97	-1.15	-2.22	3.81E-02	3.84E-01	LemA family protein
PIOMA14_RS03955	PIOMA14_I_0778		143.74	-1.15	-2.21	3.79E-02	3.84E-01	DUF4922 domain-containing protein
PIOMA14_RS13695	PIOMA14_II_0710		123.12	-1.15	-2.21	4.42E-02	4.01E-01	deoxynucleoside kinase
PIOMA14_RS08360	PIOMA14_I_1653		68.26	-1.13	-2.19	3.48E-02	3.69E-01	chromate transporter
PIOMA14_RS07605	PIOMA14_I_1498		55.67	-1.13	-2.19	4.54E-02	4.04E-01	TrkH family potassium uptake protein
PIOMA14_RS06365	PIOMA14_I_1247		74.25	-1.12	-2.17	6.57E-02	4.65E-01	reverse transcriptase-like protein
PIOMA14_RS04135	PIOMA14_I_0811		485.03	-1.11	-2.16	8.70E-02	5.28E-01	hypothetical protein, gliding motility lipoprotein GldH
PIOMA14_RS02760	PIOMA14_I_0552		52.07	-1.10	-2.14	6.56E-02	4.65E-01	HD domain-containing protein
PIOMA14_RS10930	PIOMA14_II_0137		88.34	-1.09	-2.14	5.60E-02	4.37E-01	VWA domain-containing protein
PIOMA14_RS03925	PIOMA14_I_0773		156.13	-1.09	-2.13	5.08E-02	4.29E-01	DUF4494 domain-containing protein
PIOMA14_RS03145	PIOMA14_I_0627		46.23	-1.09	-2.12	7.70E-02	5.05E-01	DNA/RNA non-specific endonuclease
PIOMA14_RS00545	PIOMA14_I_0109		120.90	-1.08	-2.12	5.50E-02	4.37E-01	cytidylate kinase-like family protein
PIOMA14_RS03870	PIOMA14_I_0762		191.76	-1.08	-2.11	5.05E-02	4.29E-01	ZIP family metal transporter
PIOMA14_RS01075	PIOMA14_I_0216		26.81	-1.08	-2.11	2.03E-02	2.90E-01	RecQ family ATP-dependent DNA helicase
PIOMA14_RS03965	PIOMA14_I_0780		71.64	-1.08	-2.11	3.83E-02	3.84E-01	MFS transporter
PIOMA14_RS03295	PIOMA14_I_0657		127.77	-1.07	-2.11	9.84E-02	5.34E-01	trimeric intracellular cation channel family protein
PIOMA14_RS10065	PIOMA14_I_1974	folE	160.17	-1.07	-2.10	6.68E-02	4.71E-01	GTP cyclohydrolase I FolE
PIOMA14_RS00880	PIOMA14_I_0179		46.54	-1.07	-2.10	2.90E-02	3.57E-01	F0F1 ATP synthase subunit beta
PIOMA14_RS05685	PIOMA14_I_1116		49.96	-1.06	-2.08	6.77E-02	4.73E-01	PD40 domain-containing protein
PIOMA14_RS03840	PIOMA14_I_0755		215.56	-1.05	-2.07	5.71E-02	4.37E-01	M6 family metalloprotease domain-containing protein
PIOMA14_RS08905	PIOMA14_I_1759		160.18	-1.04	-2.06	4.11E-02	3.93E-01	hypothetical protein
PIOMA14_RS03920	PIOMA14_I_0772		125.89	-1.03	-2.05	7.27E-02	4.85E-01	ABC transporter permease

PIOMA14_RS05780	PIOMA14_I_1133		262.25	-1.03	-2.04	8.65E-02	5.28E-01	3-deoxy-D-manno-octulosonic acid transferase, zinc finger-like domain-containing protein
PIOMA14_RS01755	PIOMA14_I_0353		45.29	-1.02	-2.03	7.84E-02	5.05E-01	response regulator transcription factor, AbgT family transporter
PIOMA14_RS00920	PIOMA14_I_0187		224.36	-1.02	-2.03	3.29E-02	3.69E-01	F0F1 ATP synthase subunit gamma
PIOMA14_RS11430	PIOMA14_II_0226		472.39	-1.02	-2.02	8.17E-02	5.15E-01	site-specific integrase
PIOMA14_RS12420	PIOMA14_II_0432		80.44	-1.02	-2.02	8.90E-02	5.28E-01	TonB-dependent receptor

[1] Gene expression level

[2] Ratio of OxyR mut vs WT

[3] P value <0.01

[4] FDR

Supplementary Table S6. Genes upregulated in *P. intermedia* OMA14 OxyR mutant (2 ≥ fold, P≤0.1)

locus tag	old locus tag	gene name	Max group mean[1]	Log_2 fold change[2]	Fold change[2]	P-value[3]	FDR p-value[4]	product (Genome (CDS))
PIOMA14_RS05040	PIOMA14_I_0995		3.88	7.44	174.05	1.78E-03	7.46E-02	hypothetical protein
PIOMA14_RS05245	PIOMA14_I_1035		4.13	5.73	52.92	1.71E-02	2.69E-01	site-specific integrase
PIOMA14_RS06470	PIOMA14_I_1270		3.57	5.15	35.61	3.44E-02	3.69E-01	toprim domain-containing protein
PIOMA14_RS12180	PIOMA14_II_0393		2.90	5.15	35.61	3.44E-02	3.69E-01	hypothetical protein
PIOMA14_RS12570	PIOMA14_II_0462		5.68	5.15	35.61	3.44E-02	3.69E-01	hypothetical protein
PIOMA14_RS06735	PIOMA14_I_1320		4.27	4.75	26.96	5.43E-02	4.37E-01	thiamine phosphate synthase
PIOMA14_RS08490	PIOMA14_I_1679		2.90	4.75	26.96	5.43E-02	4.37E-01	AAA family ATPase
PIOMA14_RS04970	PIOMA14_I_0981		8.77	4.75	26.96	5.43E-02	4.37E-01	helix-turn-helix domain-containing
PIOMA14_RS02515	PIOMA14_I_0506		7.37	4.75	26.85	5.47E-02	4.37E-01	hypothetical protein
PIOMA14_RS06415	PIOMA14_I_1257		1.30	4.19	18.31	9.76E-02	5.34E-01	site-specific integrase, hypothetical
PIOMA14_RS05195	PIOMA14_I_1025		4.63	4.19	18.31	9.77E-02	5.34E-01	hypothetical protein
PIOMA14_RS06185	PIOMA14_I_1213		2.06	4.19	18.31	9.77E-02	5.34E-01	ATP-dependent DNA helicase RecG
PIOMA14_RS08460	PIOMA14_I_1673		1.11	4.19	18.31	9.77E-02	5.34E-01	type II CRISPR RNA-guided Cas9
PIOMA14_RS12900	PIOMA14_II_0545		10.87	3.88	14.68	4.40E-03	1.34E-01	DUF1896 domain-containing protein
PIOMA14_RS11345			3.49	3.62	12.28	9.00E-03	1.97E-01	ISL3 family transposase
PIOMA14_RS01210	PIOMA14_I_0246		5.36	3.42	10.69	2.06E-04	1.67E-02	thiol-activated cytolysin family
PIOMA14_RS11155	PIOMA14_II_0177		16.04	3.32	9.96	2.17E-06	4.32E-04	restriction endonuclease subunit S
PIOMA14_RS02885	PIOMA14_I_0575		8.83	3.30	9.88	2.00E-02	2.90E-01	VOC family protein
PIOMA14_RS12940	PIOMA14_II_0554		7.39	3.30	9.88	2.00E-02	2.90E-01	DUF3872 domain-containing protein
PIOMA14_RS12120	PIOMA14_II_0379		11.13	3.30	9.88	2.00E-02	2.90E-01	hypothetical protein
PIOMA14_RS04940	PIOMA14_I_0973		4.54	3.09	8.52	5.15E-03	1.38E-01	relaxase/mobilization nuclease dom.
PIOMA14_RS11200	PIOMA14_II_0187		4.55	3.09	8.52	5.15E-03	1.38E-01	site-specific integrase
PIOMA14_RS11920	PIOMA14_II_0335		2.88	2.99	7.97	1.41E-03	6.03E-02	TonB-dependent receptor
PIOMA14_RS12185	PIOMA14_II_0394		11.07	2.91	7.54	4.89E-02	4.25E-01	DUF4372 domain-containing protein
PIOMA14_RS04980	PIOMA14_I_0983	vsr_1	5.35	2.90	7.48	4.93E-02	4.26E-01	DNA mismatch endonuclease Vsr
PIOMA14_RS14590			35.13	2.86	7.26	4.44E-03	1.34E-01	hypothetical protein
PIOMA14_RS07230	PIOMA14_I_1416		34.01	2.85	7.23	2.38E-08	1.02E-05	leucine-rich repeat domain-contai.
PIOMA14_RS01980	PIOMA14_I_0392		6.63	2.83	7.13	1.25E-02	2.43E-01	sugar transferase
PIOMA14_RS06150	PIOMA14_I_1205		554.64	2.83	7.11	5.82E-11	3.73E-08	hypothetical protein
PIOMA14_RS05240	PIOMA14_I_1034		6.15	2.79	6.91	1.03E-03	5.25E-02	hypothetical protein, site-specific
PIOMA14_RS09425	PIOMA14_I_1859		4.79	2.59	6.01	8.42E-03	1.88E-01	relaxase/mobilization nuclease domai
PIOMA14_RS06465			2.68	2.52	5.74	3.24E-02	3.69E-01	virulence protein
PIOMA14_RS01145	PIOMA14_I_0232		2.85	2.52	5.74	3.24E-02	3.69E-01	hypothetical protein, arginase family
PIOMA14_RS12895	PIOMA14_II_0544		2.76	2.52	5.74	3.24E-02	3.69E-01	site-specific integrase
PIOMA14_RS12015	PIOMA14_II_0359		16.52	2.38	5.19	3.87E-02	3.85E-01	hypothetical protein
PIOMA14_RS09830	PIOMA14_I_1934		46.94	2.35	5.11	4.03E-04	2.59E-02	nucleoside deaminase
PIOMA14_RS00055	PIOMA14_I_0011		82.87	2.33	5.03	1.14E-06	2.92E-04	carboxypeptidase-like regulator SAM
PIOMA14_RS01220	PIOMA14_I_0248		30.81	2.32	5.00	3.08E-03	1.04E-01	hypothetical protein
PIOMA14_RS11150	PIOMA14_II_0176		23.14	2.32	5.00	1.27E-03	5.92E-02	restriction endonuclease subunit S
PIOMA14_RS05580	PIOMA14_I_1101		18.23	2.28	4.84	2.43E-03	8.94E-02	hypothetical protein
PIOMA14_RS00725	PIOMA14_I_0146		16.54	2.21	4.64	1.53E-02	2.60E-01	hypothetical protein
PIOMA14_RS01215	PIOMA14_I_0247		11.34	2.21	4.64	1.47E-02	2.60E-01	DNA-binding protein
PIOMA14_RS11395	PIOMA14_II_0220		200.83	2.20	4.59	2.19E-06	4.32E-04	choice-of-anchor J domain-containing
PIOMA14_RS08785	PIOMA14_I_1738		18.27	2.18	4.54	4.66E-04	2.85E-02	ATPase
PIOMA14_RS13620	PIOMA14_II_0693		6.73	2.12	4.34	8.87E-02	5.28E-01	HlyD family secretion protein
PIOMA14_RS01540	PIOMA14_I_0313		4.34	2.12	4.34	8.87E-02	5.28E-01	conjugal transfer protein TraO, topr.
PIOMA14_RS06475	PIOMA14_I_1271		5.52	2.12	4.34	8.87E-02	5.28E-01	DUF3408 domain-containing protein
PIOMA14_RS11190			6.88	2.12	4.34	8.87E-02	5.28E-01	helix-turn-helix domain-containing
PIOMA14_RS11165	PIOMA14_II_0179		4.83	2.12	4.34	8.88E-02	5.28E-01	hypothetical protein
PIOMA14_RS08145			43.40	2.02	4.07	1.41E-03	6.03E-02	leucine-rich repeat protein
PIOMA14_RS07375	PIOMA14_I_1448		5.80	2.02	4.04	5.79E-02	4.38E-01	hypothetical protein
PIOMA14_RS00760	PIOMA14_I_0153		122.77	1.98	3.94	2.98E-02	3.60E-01	hypothetical protein
PIOMA14_RS12190			13.66	1.96	3.88	3.82E-02	3.84E-01	transposase
PIOMA14_RS12735	PIOMA14_II_0501		3.40	1.95	3.88	3.82E-02	3.84E-01	site-specific integrase
PIOMA14_RS07765	PIOMA14_I_1531		9.02	1.94	3.83	1.41E-02	2.58E-01	IS4 family transposase
PIOMA14_RS01150	PIOMA14_I_0233		2.46	1.92	3.78	2.54E-02	3.32E-01	TonB-dependent receptor family protein
PIOMA14_RS08450	PIOMA14_I_1671	cas2	17.04	1.92	3.77	3.38E-02	3.69E-01	CRISPR-associated endonuclease Cas2
PIOMA14_RS14090	PIOMA14_II_0783		16.54	1.92	3.77	3.37E-02	3.69E-01	hypothetical protein
PIOMA14_RS13215	PIOMA14_II_0618		759.43	1.92	3.77	2.42E-02	3.26E-01	hypothetical protein
PIOMA14_RS05110	PIOMA14_I_1008		9.55	1.91	3.75	1.82E-03	7.52E-02	DUF935 family protein
PIOMA14_RS08960	PIOMA14_I_1771		364.09	1.90	3.74	4.04E-04	2.59E-02	hypothetical protein
PIOMA14_RS00670	PIOMA14_I_0135		30.63	1.90	3.73	2.28E-03	8.85E-02	DUF1320 family protein
PIOMA14_RS13155	PIOMA14_II_0606		2112.91	1.89	3.71	9.80E-05	9.30E-03	hypothetical protein
PIOMA14_RS00595	PIOMA14_I_0119		44.11	1.89	3.70	1.90E-03	7.52E-02	hypothetical protein
PIOMA14_RS02265	PIOMA14_I_0449		57.49	1.88	3.69	1.41E-02	2.58E-01	TetR/AcrR family transcriptional regulator
PIOMA14_RS06055	PIOMA14_I_1185		75.52	1.88	3.68	2.53E-02	3.32E-01	BlaI/MecI/CopY family transcriptional regulator
PIOMA14_RS02235	PIOMA14_I_0443		16.65	1.86	3.64	1.08E-03	5.34E-02	ATP-binding protein
PIOMA14_RS12330	PIOMA14_II_0417		128.46	1.85	3.61	5.22E-04	3.11E-02	ParA family protein
PIOMA14_RS13015	PIOMA14_II_0573		32.04	1.84	3.59	2.47E-02	3.26E-01	hypothetical protein
PIOMA14_RS02895	PIOMA14_I_0577		664.34	1.84	3.57	1.39E-03	6.03E-02	Crp/Fnr family transcriptional regulator
PIOMA14_RS06165	PIOMA14_I_1208	vsr_2	97.10	1.82	3.54	5.29E-03	1.38E-01	DNA mismatch endonuclease Vsr
PIOMA14_RS00865	PIOMA14_I_0177		11.97	1.81	3.50	5.43E-03	1.39E-01	oligosaccharide flippase family protein, glycosyltransferase family 2 protein
PIOMA14_RS01380	PIOMA14_I_0280		6.37	1.80	3.48	3.03E-03	1.04E-01	type IA DNA topoisomerase
PIOMA14_RS05575	PIOMA14_I_1100		24.31	1.78	3.44	5.09E-03	1.38E-01	hypothetical protein
PIOMA14_RS02960	PIOMA14_I_0589		124.67	1.71	3.28	4.46E-02	4.01E-01	GlsB/YeaQ/YmgE family stress response membrane
PIOMA14_RS03615	PIOMA14_I_0713		3.61	1.69	3.24	3.38E-02	3.69E-01	TonB-dependent receptor
PIOMA14_RS14125	PIOMA14_II_0790		105.31	1.68	3.21	6.44E-02	4.65E-01	hypothetical protein
PIOMA14_RS07955	PIOMA14_I_1582		4.06	1.67	3.18	5.76E-02	4.38E-01	hypothetical protein, site-specific integrase

PIOMA14_RS12855	PIOMA14_II_0534		16.38	1.67	3.18	5.70E-02	4.37E-01	DUF3853 family protein, AAA family ATPase
PIOMA14_RS11160	PIOMA14_II_0178		5.49	1.67	3.18	4.34E-02	4.01E-01	site-specific integrase
PIOMA14_RS06720	PIOMA14_I_1317		6.88	1.66	3.16	6.38E-02	4.65E-01	bifunctional hydroxymethylpyrimidine kinase/phosphomethylpyrimidine kinase, thiamine phosphate synthase
PIOMA14_RS13630	PIOMA14_II_0696		1.81	1.66	3.16	6.39E-02	4.65E-01	TonB-dependent receptor
PIOMA14_RS01085	PIOMA14_I_0217		3.19	1.64	3.12	9.87E-02	5.34E-01	relaxase/mobilization nuclease domain-containing protein, hypothetical protein
PIOMA14_RS13680	PIOMA14_II_0708		3.21	1.64	3.12	9.87E-02	5.34E-01	hypothetical protein, relaxase/mobilization nuclease domain-containing
PIOMA14_RS08110	PIOMA14_I_1605		16.95	1.64	3.12	9.89E-02	5.34E-01	DUF4250 domain-containing protein
PIOMA14_RS12085	PIOMA14_II_0372		57.31	1.64	3.11	3.90E-02	3.86E-01	phage virion morphogenesis protein, hypothetical protein
PIOMA14_RS13120	PIOMA14_II_0598		28.64	1.63	3.10	4.72E-03	1.38E-01	hypothetical protein
PIOMA14_RS01340	PIOMA14_I_0271		20.60	1.62	3.06	5.09E-02	4.29E-01	site-specific integrase

[1] Gene expression level

[2] Ratio of OxyR mut vs WT

[3] P value <0.01

[4] FDR

Supplementary Table S7. Genes downregulated in *P. intermedia* OMA14 OxyR mutant in iron deplete conditions (2 ≥ fold, P≤0.1)

locus tag	old locus tag	gene name	[1]Max group mean	[2]Log_2 fold change	[2]Fold change	[3]P-value	[4]FDR p-value	product (Genome (CDS))
PIOMA14_RS00365	PIOMA14_I_0073	OxyR	115.40	-8.70	-417.13	2.64E-03	4.93E-02	hydrogen peroxide-inducible genes activator
PIOMA14_RS00355	PIOMA14_I_0070	ahpC	2366.28	-8.67	-406.28	0.00E+00	0.00E+00	peroxiredoxin
PIOMA14_RS00350	PIOMA14_I_0069	ahpF	3512.44	-7.74	-214.06	4.44E-11	1.85E-08	alkyl hydroperoxide reductase subunit F
PIOMA14_RS10430	PIOMA14_II_0050		24.42	-5.45	-43.74	5.97E-02	3.05E-01	hypothetical protein
PIOMA14_RS01990	PIOMA14_I_0394		6.68	-5.00	-32.07	8.51E-02	3.72E-01	glycosyltransferase family 4 protein
PIOMA14_RS02020	PIOMA14_I_0401		13.84	-5.00	-32.07	8.51E-02	3.72E-01	serine acetyltransferase
PIOMA14_RS02545	PIOMA14_I_0510		29.23	-4.80	-27.79	6.88E-04	1.70E-02	GNAT family N-acetyltransferase, glycosyltransferase family 2 protein, hypothetical protein
PIOMA14_RS09985	PIOMA14_I_1962		1119.10	-4.37	-20.64	0.00E+00	0.00E+00	DNA starvation/stationary phase protection protein
PIOMA14_RS09980	PIOMA14_I_1961	folK	51.18	-3.22	-9.32	3.04E-03	5.39E-02	2-amino-4-hydroxy-6-hydroxymethyldihydropteridine diphosphokinase
PIOMA14_RS09975	PIOMA14_I_1960	queA	63.09	-3.17	-9.02	1.02E-06	8.25E-05	tRNA pseudouridine(55) synthase TruB, tRNA preQ1(34) S-adenosylmethionine ribosyltransferase-isomerase QueA
PIOMA14_RS02000	PIOMA14_I_0397		22.63	-3.15	-8.88	3.31E-03	5.58E-02	glycosyltransferase family 2 protein
PIOMA14_RS08395	PIOMA14_I_1659		34.41	-3.15	-8.88	2.94E-03	5.29E-02	nicotinate-nucleotide adenylyltransferase
PIOMA14_RS12605	PIOMA14_II_0469		24.20	-3.15	-8.87	2.27E-02	1.78E-01	DUF3872 domain-containing protein
PIOMA14_RS03720	PIOMA14_I_0734		34.51	-3.01	-8.05	3.23E-02	2.16E-01	DUF721 domain-containing protein, DNA replication and repair protein RecF
PIOMA14_RS12210	PIOMA14_II_0399		164.35	-3.00	-8.00	9.37E-10	1.95E-07	hypothetical protein
PIOMA14_RS11625	PIOMA14_II_0276		26.01	-2.83	-7.13	1.55E-02	1.40E-01	DUF4141 domain-containing protein
PIOMA14_RS11415	PIOMA14_II_0224		243.86	-2.82	-7.05	1.63E-12	8.13E-10	DUF2723 domain-containing protein
PIOMA14_RS10745	PIOMA14_II_0104	nrdG	106.56	-2.78	-6.86	6.63E-04	1.66E-02	anaerobic ribonucleoside-triphosphate reductase activating protein
PIOMA14_RS11555	PIOMA14_II_0258		78.39	-2.69	-6.47	6.49E-03	8.25E-02	hypothetical protein
PIOMA14_RS02005	PIOMA14_I_0398		11.20	-2.54	-5.81	1.49E-02	1.38E-01	EpsG family protein
PIOMA14_RS10170	PIOMA14_I_1995	folB	18.28	-2.51	-5.71	7.20E-02	3.43E-01	dihydroneopterin aldolase
PIOMA14_RS12695	PIOMA14_II_0490		868.01	-2.51	-5.70	2.26E-04	7.52E-03	toxin PIN
PIOMA14_RS04685	PIOMA14_I_0924		17.32	-2.47	-5.54	1.07E-02	1.10E-01	o-succinylbenzoate synthase
PIOMA14_RS04860	PIOMA14_I_0956		11.69	-2.43	-5.37	2.07E-02	1.67E-01	porin
PIOMA14_RS09180	PIOMA14_I_1810		11.76	-2.43	-5.37	2.07E-02	1.67E-01	S41 family peptidase, DUF3316 domain-containing protein
PIOMA14_RS03550	PIOMA14_I_0698	carB	112.54	-2.37	-5.19	2.55E-05	1.32E-03	glutamine-hydrolyzing carbamoyl-phosphate synthase small subunit, carbamoyl-phosphate synthase (glutamine-hydrolyzing) large subunit
PIOMA14_RS04385	PIOMA14_I_0859		22.88	-2.37	-5.17	1.08E-02	1.11E-01	calcium/sodium antiporter
PIOMA14_RS03370	PIOMA14_I_0668		304.86	-2.33	-5.03	1.74E-04	5.96E-03	3-methyl-2-oxobutanoate dehydrogenase subunit VorB
PIOMA14_RS11405	PIOMA14_II_0222	queG	130.70	-2.32	-4.98	4.62E-07	4.12E-05	tRNA epoxyqueuosine(34) reductase QueG, polysaccharide deacetylase family protein
PIOMA14_RS03365	PIOMA14_I_0667		110.84	-2.31	-4.94	7.52E-03	8.72E-02	4Fe-4S binding protein
PIOMA14_RS00890	PIOMA14_I_0181		26.17	-2.30	-4.93	2.98E-02	2.09E-01	hypothetical protein, F0F1 ATP synthase subunit A
PIOMA14_RS11570	PIOMA14_II_0262		936.29	-2.29	-4.88	1.89E-03	3.80E-02	toxin PIN
PIOMA14_RS04015	PIOMA14_I_0789		173.06	-2.27	-4.83	1.43E-05	7.94E-04	DUF1015 domain-containing protein
PIOMA14_RS03545	PIOMA14_I_0697	carA	81.75	-2.26	-4.78	1.17E-04	4.42E-03	glutamine-hydrolyzing carbamoyl-phosphate synthase small subunit, carbamoyl-phosphate synthase (glutamine-hydrolyzing) large subunit
PIOMA14_RS01970	PIOMA14_I_0390		18.01	-2.23	-4.71	8.66E-03	9.70E-02	nucleotidyltransferase family protein
PIOMA14_RS12205	PIOMA14_II_0398		158.74	-2.22	-4.65	1.39E-04	4.95E-03	MotA/TolQ/ExbB proton channel family protein
PIOMA14_RS07870	PIOMA14_I_1551		144.83	-2.20	-4.61	9.71E-06	5.64E-04	hypothetical protein
PIOMA14_RS12700	PIOMA14_II_0491		505.20	-2.19	-4.57	1.91E-08	2.65E-06	peptidase M26
PIOMA14_RS09085	PIOMA14_I_1790		16.72	-2.17	-4.49	4.16E-02	2.52E-01	hypothetical protein
PIOMA14_RS07855	PIOMA14_I_1548		1093.90	-2.17	-4.49	5.91E-11	2.11E-08	Omp28-related outer membrane protein
PIOMA14_RS04885	PIOMA14_I_0961		25.81	-2.11	-4.33	4.13E-02	2.51E-01	hypothetical protein
PIOMA14_RS03275	PIOMA14_I_0653		96.15	-2.11	-4.32	3.88E-04	1.17E-02	HAD family hydrolase
PIOMA14_RS11610	PIOMA14_II_0273		88.82	-2.11	-4.31	1.56E-02	1.40E-01	DUF4134 domain-containing protein
PIOMA14_RS03220	PIOMA14_I_0643		26.57	-2.08	-4.24	1.54E-02	1.40E-01	copper homeostasis protein CutC
PIOMA14_RS11565	PIOMA14_II_0261		503.95	-2.06	-4.18	3.92E-09	7.54E-07	peptidase M26
PIOMA14_RS11220	PIOMA14_II_0190		46.79	-2.03	-4.07	1.18E-03	2.53E-02	leucine-rich repeat domain-containing protein
PIOMA14_RS07405	PIOMA14_I_1455		14.41	-2.02	-4.06	6.01E-02	3.05E-01	agmatine deiminase family protein
PIOMA14_RS12040	PIOMA14_II_0364		19.46	-2.02	-4.06	6.01E-02	3.05E-01	N-acetylmuramoyl-L-alanine amidase
PIOMA14_RS02015	PIOMA14_I_0400		9.18	-2.02	-4.06	6.11E-02	3.09E-01	glycosyltransferase
PIOMA14_RS01305	PIOMA14_I_0264	ubiE	17.67	-2.01	-4.03	2.99E-02	2.09E-01	bifunctional demethylmenaquinone methyltransferase/2-methoxy-6-polyprenyl-1,4-benzoquinol methylase UbiE
PIOMA14_RS04075	PIOMA14_I_0800		1024.56	-2.00	-3.99	9.03E-09	1.41E-06	DEAD/DEAH box helicase
PIOMA14_RS03835	PIOMA14_I_0754		415.91	-1.99	-3.98	7.99E-05	3.22E-03	chitobiase/beta-hexosaminidase C-terminal domain-containing protein
PIOMA14_RS03940	PIOMA14_I_0776		169.45	-1.94	-3.84	1.20E-04	4.47E-03	DNA-3-methyladenine glycosylase I
PIOMA14_RS02045	PIOMA14_I_0405		18.71	-1.94	-3.83	1.43E-02	1.34E-01	nucleotide sugar dehydrogenase
PIOMA14_RS07860	PIOMA14_I_1549		112.04	-1.92	-3.79	3.55E-05	1.71E-03	C10 family peptidase
PIOMA14_RS12805	PIOMA14_II_0518		11.30	-1.90	-3.72	3.58E-02	2.29E-01	nucleotidyl transferase AbiEii/AbiGii toxin family protein, hypothetical protein
PIOMA14_RS11560	PIOMA14_II_0259		767.85	-1.88	-3.68	1.93E-08	2.65E-06	hypothetical protein
PIOMA14_RS11595	PIOMA14_II_0270		63.31	-1.86	-3.62	1.00E-02	1.07E-01	conjugal transfer protein TraA, DUF3408 domain-containing protein
PIOMA14_RS11505	PIOMA14_II_0245		12.76	-1.85	-3.62	8.80E-02	3.73E-01	DUF3408 domain-containing protein
PIOMA14_RS07850	PIOMA14_I_1547		134.64	-1.84	-3.58	3.41E-03	5.58E-02	TlpA family protein disulfide reductase
PIOMA14_RS02540	PIOMA14_I_0509		148.23	-1.82	-3.54	2.04E-02	1.67E-01	GNAT family N-acetyltransferase, glycosyltransferase family 2 protein
PIOMA14_RS03335	PIOMA14_I_0662	ligA	643.39	-1.81	-3.52	1.17E-10	3.24E-08	NAD-dependent DNA ligase LigA
PIOMA14_RS11410	PIOMA14_II_0223		98.10	-1.81	-3.51	3.23E-03	5.58E-02	tRNA epoxyqueuosine(34) reductase QueG, polysaccharide deacetylase family protein
PIOMA14_RS03725	PIOMA14_I_0735	recF	38.54	-1.80	-3.49	4.38E-03	6.32E-02	DUF721 domain-containing protein, DNA replication and repair protein RecF
PIOMA14_RS03930	PIOMA14_I_0774		239.38	-1.79	-3.45	2.54E-03	4.85E-02	YggS family pyridoxal phosphate-dependent enzyme
PIOMA14_RS11095	PIOMA14_II_0165		8.25	-1.77	-3.42	5.18E-02	2.77E-01	AAA family ATPase
PIOMA14_RS09690	PIOMA14_I_1907		12.15	-1.77	-3.42	5.18E-02	2.77E-01	DMT family transporter
PIOMA14_RS00215	PIOMA14_I_0045		211.38	-1.77	-3.41	1.29E-04	4.72E-03	imidazolonepropionase, histidine ammonia-lyase
PIOMA14_RS12705	PIOMA14_II_0493		787.25	-1.76	-3.39	1.26E-07	1.43E-05	hypothetical protein

PIOMA14_RS11605	PIOMA14_II_0272		60.10	-1.74	-3.35	1.15E-02	1.16E-01	hypothetical protein
PIOMA14_RS03785	PIOMA14_I_0745	guaA	272.25	-1.74	-3.35	4.56E-08	5.70E-06	glutamine-hydrolyzing GMP synthase
PIOMA14_RS00880	PIOMA14_I_0179		44.50	-1.74	-3.33	1.19E-03	2.54E-02	F0F1 ATP synthase subunit beta
PIOMA14_RS04235	PIOMA14_I_0831		71.59	-1.74	-3.33	9.95E-07	8.25E-05	SLC13/DASS family transporter
PIOMA14_RS03375	PIOMA14_I_0669		2895.33	-1.73	-3.32	2.45E-05	1.30E-03	2-oxoglutarate oxidoreductase
PIOMA14_RS10345	PIOMA14_II_0032		12.06	-1.73	-3.31	7.24E-02	3.44E-01	hypothetical protein
PIOMA14_RS02365	PIOMA14_I_0474		123.74	-1.73	-3.31	9.13E-06	5.43E-04	hemagglutinin
PIOMA14_RS12710	PIOMA14_II_0494		89.70	-1.72	-3.31	3.74E-02	2.34E-01	hypothetical protein
PIOMA14_RS03070	PIOMA14_I_0612	gcvT	114.24	-1.72	-3.30	4.66E-05	2.01E-03	glycine cleavage system aminomethyltransferase GcvT
PIOMA14_RS02375	PIOMA14_I_0476		124.29	-1.70	-3.25	3.66E-05	1.72E-03	hypothetical protein
PIOMA14_RS03745	PIOMA14_I_0738	secG	44.99	-1.70	-3.25	4.23E-02	2.53E-01	hypothetical protein, preprotein translocase subunit SecG
PIOMA14_RS07675	PIOMA14_I_1510		42.10	-1.70	-3.25	5.06E-02	2.76E-01	site-specific integrase, hypothetical protein
PIOMA14_RS09200	PIOMA14_I_1814		109.61	-1.70	-3.24	1.98E-03	3.93E-02	FKBP-type peptidyl-prolyl cis-trans isomerase
PIOMA14_RS03935	PIOMA14_I_0775		242.23	-1.68	-3.20	3.48E-13	2.18E-10	NAD(P)/FAD-dependent oxidoreductase
PIOMA14_RS02595	PIOMA14_I_0521	nifJ	21.87	-1.67	-3.19	1.98E-03	3.93E-02	pyruvate:ferredoxin (flavodoxin) oxidoreductase
PIOMA14_RS01280	PIOMA14_I_0259		31.98	-1.66	-3.17	5.57E-03	7.46E-02	HD domain-containing protein
PIOMA14_RS06695	PIOMA14_I_1312		55.36	-1.66	-3.17	5.08E-03	7.07E-02	hypothetical protein
PIOMA14_RS00800	PIOMA14_I_0164		56.07	-1.66	-3.15	9.23E-03	1.02E-01	PorT family protein
PIOMA14_RS07535	PIOMA14_I_1484	radC	41.85	-1.64	-3.13	1.99E-02	1.65E-01	DNA repair protein RadC
PIOMA14_RS03540	PIOMA14_I_0696		486.80	-1.64	-3.12	7.00E-09	1.17E-06	amidophosphoribosyltransferase
PIOMA14_RS03775	PIOMA14_I_0743		649.87	-1.62	-3.08	2.95E-10	6.69E-08	hypothetical protein, phosphotransferase
PIOMA14_RS02400	PIOMA14_I_0483		54.61	-1.62	-3.08	2.46E-02	1.87E-01	conjugal transfer protein TraA, DUF3408 domain-containing protein
PIOMA14_RS12620	PIOMA14_II_0472	traM_4	39.43	-1.61	-3.06	7.62E-03	8.72E-02	conjugative transposon protein TraM, hypothetical protein
PIOMA14_RS04645	PIOMA14_I_0914		39.65	-1.61	-3.06	8.62E-02	3.73E-01	hypothetical protein, site-specific integrase
PIOMA14_RS04020	PIOMA14_I_0790		512.74	-1.61	-3.05	3.86E-03	5.88E-02	YigZ family protein
PIOMA14_RS12640	PIOMA14_II_0476		30.85	-1.61	-3.04	4.64E-02	2.66E-01	DUF4141 domain-containing protein
PIOMA14_RS01260	PIOMA14_I_0255	miaA_1	167.77	-1.60	-3.04	5.33E-03	7.27E-02	tRNA (adenosine(37)-N6)-dimethylallyltransferase MiaA
PIOMA14_RS03770	PIOMA14_I_0742		916.65	-1.59	-3.02	6.42E-04	1.62E-02	hypothetical protein, phosphotransferase
PIOMA14_RS10165	PIOMA14_I_1974		159.74	-1.57	-2.97	3.89E-07	3.89E-05	adenosylcobalamin-dependent ribonucleoside-diphosphate reductase
PIOMA14_RS02590	PIOMA14_I_0520		58.26	-1.56	-2.94	7.29E-03	8.55E-02	ATP-binding protein
PIOMA14_RS08830	PIOMA14_I_1746		63.79	-1.56	-2.94	3.04E-03	5.39E-02	hypothetical protein
PIOMA14_RS07840	PIOMA14_I_1545		35.16	-1.54	-2.91	3.53E-02	2.29E-01	Omp28 family outer membrane lipoprotein
PIOMA14_RS03900	PIOMA14_I_0769	truA	95.61	-1.54	-2.91	4.40E-03	6.32E-02	tRNA pseudouridine(38-40) synthase TruA
PIOMA14_RS10490	PIOMA14_II_0063		206.49	-1.54	-2.91	1.04E-03	2.29E-02	NADH:ubiquinone reductase (Na(+)-transporting) subunit F
PIOMA14_RS09090	PIOMA14_I_1791		26.55	-1.54	-2.90	4.99E-02	2.75E-01	hypothetical protein
PIOMA14_RS02370	PIOMA14_I_0475		122.05	-1.54	-2.90	1.35E-04	4.89E-03	peptidase M26
PIOMA14_RS08680	PIOMA14_I_1718	cas9	22.47	-1.53	-2.89	7.56E-03	8.72E-02	type II CRISPR RNA-guided endonuclease Cas9
PIOMA14_RS03345	PIOMA14_I_0663		19.86	-1.52	-2.87	9.03E-02	3.78E-01	DUF4359 domain-containing protein
PIOMA14_RS10740	PIOMA14_II_0103	nrdD	1282.85	-1.52	-2.87	2.48E-03	4.79E-02	anaerobic ribonucleoside-triphosphate reductase
PIOMA14_RS09735	PIOMA14_I_1917	mrdA	43.77	-1.52	-2.86	4.20E-04	1.22E-02	rod shape-determining protein MreD, penicillin-binding protein 2
PIOMA14_RS03685	PIOMA14_I_0727		137.92	-1.50	-2.83	1.44E-02	1.34E-01	glycosyltransferase family 2 protein
PIOMA14_RS02765	PIOMA14_I_0553		190.38	-1.50	-2.82	1.04E-03	2.29E-02	nucleoside deaminase
PIOMA14_RS12690	PIOMA14_II_0488		36.93	-1.48	-2.80	7.11E-03	8.43E-02	YWFCY domain-containing protein
PIOMA14_RS03535	PIOMA14_I_0695	glmS	288.31	-1.47	-2.77	3.82E-03	5.87E-02	glutamine--fructose-6-phosphate transaminase (isomerizing)
PIOMA14_RS08820	PIOMA14_I_1744		55.04	-1.47	-2.77	5.11E-04	1.36E-02	RagB/SusD family nutrient uptake outer membrane protein
PIOMA14_RS13240	PIOMA14_II_0624		41.40	-1.46	-2.75	3.37E-02	2.22E-01	DNA/RNA non-specific endonuclease
PIOMA14_RS03710	PIOMA14_I_0732		108.48	-1.46	-2.75	9.70E-03	1.05E-01	dCMP deaminase family protein, S41 family peptidase
PIOMA14_RS04065	PIOMA14_I_0798		282.38	-1.45	-2.74	1.37E-02	1.30E-01	thymidine kinase
PIOMA14_RS01060	PIOMA14_I_0213		45.78	-1.45	-2.73	5.37E-03	7.28E-02	aminopeptidase P N-terminal domain-containing protein
PIOMA14_RS03660	PIOMA14_I_0725		162.80	-1.45	-2.73	2.36E-03	4.60E-02	gliding motility protein GldB
PIOMA14_RS05685	PIOMA14_I_1116		44.14	-1.45	-2.73	7.13E-03	8.43E-02	PD40 domain-containing protein
PIOMA14_RS03050	PIOMA14_I_0608		200.18	-1.44	-2.71	1.52E-03	3.18E-02	dihydrolipoyl dehydrogenase
PIOMA14_RS12655	PIOMA14_II_0479		82.27	-1.43	-2.70	7.36E-02	3.46E-01	DUF4134 domain-containing protein
PIOMA14_RS04120	PIOMA14_I_0808		400.54	-1.41	-2.66	8.19E-07	7.05E-05	insulinase family protein
PIOMA14_RS01400	PIOMA14_I_0284		31.99	-1.41	-2.66	7.49E-03	8.72E-02	outer membrane beta-barrel protein
PIOMA14_RS09775	PIOMA14_I_1925		155.77	-1.41	-2.65	2.94E-03	5.29E-02	dipeptidase
PIOMA14_RS00385	PIOMA14_I_0077	yidC	66.74	-1.39	-2.63	3.21E-03	5.58E-02	membrane protein insertase YidC
PIOMA14_RS12685	PIOMA14_II_0487		14.85	-1.39	-2.62	6.79E-02	3.27E-01	hypothetical protein, relaxase/mobilization nuclease domain-containing protein
PIOMA14_RS01300	PIOMA14_I_0263		14.60	-1.39	-2.61	9.29E-02	3.81E-01	shikimate dehydrogenase
PIOMA14_RS00905	PIOMA14_I_0184	atpF	160.61	-1.38	-2.60	5.51E-03	7.44E-02	F0F1 ATP synthase subunit B
PIOMA14_RS03695	PIOMA14_I_0729		138.87	-1.37	-2.59	4.60E-04	1.26E-02	Crp/Fnr family transcriptional regulator
PIOMA14_RS03850	PIOMA14_I_0757		816.96	-1.36	-2.57	6.68E-03	8.35E-02	C10 family peptidase
PIOMA14_RS03490	PIOMA14_I_0687		18.09	-1.36	-2.57	8.56E-02	3.72E-01	DMT family transporter
PIOMA14_RS12625	PIOMA14_II_0473		52.94	-1.36	-2.57	9.20E-02	3.80E-01	conjugative transposon protein TraM, hypothetical protein
PIOMA14_RS10510	PIOMA14_II_0067		201.68	-1.35	-2.56	3.83E-03	5.87E-02	NADH:ubiquinone reductase (Na(+)-transporting) subunit B
PIOMA14_RS00920	PIOMA14_I_0187		207.40	-1.35	-2.56	1.16E-03	2.53E-02	F0F1 ATP synthase subunit gamma
PIOMA14_RS01270	PIOMA14_I_0257		137.43	-1.35	-2.55	6.49E-03	8.25E-02	bifunctional UDP-3-O-[3-hydroxymyristoyl] N-acetylglucosamine deacetylase/3-hydroxyacyl-ACP dehydratase
PIOMA14_RS11620	PIOMA14_II_0275	traG_3	57.33	-1.34	-2.54	2.11E-02	1.69E-01	DUF4133 domain-containing protein, TraG family conjugative transposon ATPase
PIOMA14_RS10210	PIOMA14_II_0004	rpsA	745.21	-1.34	-2.53	2.59E-05	1.32E-03	30S ribosomal protein S1
PIOMA14_RS00915	PIOMA14_I_0186		88.87	-1.34	-2.53	2.11E-02	1.69E-01	F0F1 ATP synthase subunit alpha
PIOMA14_RS11575	PIOMA14_II_0264		40.90	-1.33	-2.52	7.02E-03	8.43E-02	YWFCY domain-containing protein
PIOMA14_RS04115	PIOMA14_I_0807		741.49	-1.31	-2.49	2.59E-06	1.91E-04	DUF4876 domain-containing protein
PIOMA14_RS04095	PIOMA14_I_0803		140.89	-1.30	-2.46	4.80E-03	6.77E-02	ribosomal protein L7/L12
PIOMA14_RS00380	PIOMA14_I_0076		432.28	-1.29	-2.45	4.58E-07	4.12E-05	S9 family peptidase
PIOMA14_RS06300	PIOMA14_I_1234		1027.42	-1.29	-2.45	2.05E-04	6.90E-03	adhesin
PIOMA14_RS01265	PIOMA14_I_0256	lpxA_1	38.94	-1.29	-2.44	6.53E-02	3.25E-01	acyl-ACP--UDP-N-acetylglucosamine O-acyltransferase
PIOMA14_RS02615	PIOMA14_I_0525	ruvX	147.59	-1.29	-2.44	1.20E-02	1.19E-01	Holliday junction resolvase RuvX, SPOR domain-containing protein
PIOMA14_RS04220	PIOMA14_I_0829	nudC	31.89	-1.28	-2.43	8.52E-02	3.72E-01	NAD(+) diphosphatase
PIOMA14_RS03645	PIOMA14_I_0722		244.01	-1.28	-2.43	4.29E-02	2.54E-01	hypothetical protein
PIOMA14_RS00280	PIOMA14_I_0058		39.21	-1.28	-2.43	8.76E-02	3.73E-01	hypothetical protein
PIOMA14_RS08390	PIOMA14_I_1658	gmk	26.16	-1.27	-2.41	1.03E-01	3.99E-01	guanylate kinase
PIOMA14_RS03750	PIOMA14_I_0739		63.59	-1.27	-2.40	1.12E-02	1.14E-01	hypothetical protein
PIOMA14_RS00875	PIOMA14_I_0178	pfkA	84.88	-1.27	-2.40	5.71E-03	7.54E-02	6-phosphofructokinase

PIOMA14_RS08405	PIOMA14_I_1661		28.13	-1.26	-2.39	6.00E-02	3.05E-01	glycosyltransferase
PIOMA14_RS03965	PIOMA14_I_0780		85.02	-1.25	-2.38	1.71E-02	1.49E-01	MFS transporter
PIOMA14_RS11645	PIOMA14_II_0280	traM_3	40.18	-1.25	-2.38	3.23E-02	2.16E-01	hypothetical protein, conjugative transposon protein TraM
PIOMA14_RS00375	PIOMA14_I_0075		29.57	-1.24	-2.36	1.05E-02	1.09E-01	MFS transporter
PIOMA14_RS07210	PIOMA14_I_1412		22.53	-1.23	-2.35	9.34E-02	3.82E-01	succinate dehydrogenase/fumarate reductase iron-sulfur subunit
PIOMA14_RS03030	PIOMA14_I_0604		1167.57	-1.23	-2.35	7.40E-05	3.03E-03	hypothetical protein
PIOMA14_RS02760	PIOMA14_I_0552		55.17	-1.23	-2.35	4.37E-02	2.56E-01	HD domain-containing protein
PIOMA14_RS12630	PIOMA14_II_0474	traK_4	140.93	-1.23	-2.34	3.89E-03	5.88E-02	conjugative transposon protein TraK
PIOMA14_RS02355	PIOMA14_I_0471		39.68	-1.23	-2.34	2.36E-02	1.83E-01	AraC family transcriptional regulator
PIOMA14_RS01910	PIOMA14_I_0377		368.17	-1.22	-2.34	9.86E-04	2.22E-02	M13 family metallopeptidase
PIOMA14_RS03780	PIOMA14_I_0744		413.46	-1.22	-2.33	4.78E-03	6.77E-02	nucleotidyltransferase family protein
PIOMA14_RS03025	PIOMA14_I_0603		587.45	-1.21	-2.32	5.30E-04	1.39E-02	TonB-dependent receptor
PIOMA14_RS06375	PIOMA14_I_1249		52.71	-1.21	-2.31	5.89E-03	7.70E-02	arginine--tRNA ligase
PIOMA14_RS03055	PIOMA14_I_0609	gcvPB	275.54	-1.20	-2.30	1.84E-03	3.74E-02	aminomethyl-transferring glycine dehydrogenase subunit GcvPB
PIOMA14_RS03920	PIOMA14_I_0772		96.94	-1.20	-2.29	1.41E-02	1.33E-01	ABC transporter permease
PIOMA14_RS03790	PIOMA14_I_0746	mscL	128.76	-1.20	-2.29	3.11E-02	2.14E-01	large-conductance mechanosensitive channel protein MscL
PIOMA14_RS02415	PIOMA14_I_0486		52.57	-1.19	-2.29	6.83E-02	3.28E-01	hypothetical protein
PIOMA14_RS12645	PIOMA14_II_0477	traG_4	57.63	-1.18	-2.27	4.32E-02	2.55E-01	TraG family conjugative transposon ATPase, DUF4133 domain-containing protein
PIOMA14_RS00430	PIOMA14_I_0086	wecB	59.50	-1.18	-2.26	3.28E-02	2.18E-01	UDP-N-acetylglucosamine 2-epimerase (non-hydrolyzing), glycoside hydrolase family 99-like domain-containing protein
PIOMA14_RS05215	PIOMA14_I_1029		10.70	-1.17	-2.26	7.36E-02	3.46E-01	Z1 domain-containing protein
PIOMA14_RS02730	PIOMA14_I_0546		86.15	-1.17	-2.26	6.64E-02	3.27E-01	DUF4293 domain-containing protein, DNA-directed RNA polymerase subunit omega
PIOMA14_RS03960	PIOMA14_I_0779		482.74	-1.16	-2.24	2.79E-03	5.13E-02	SpoIID/LytB domain-containing protein
PIOMA14_RS04550	PIOMA14_I_0892		264.12	-1.16	-2.24	1.39E-02	1.32E-01	NAD(P)H-dependent glycerol-3-phosphate dehydrogenase
PIOMA14_RS07455	PIOMA14_I_1467	pnp	868.60	-1.16	-2.23	5.55E-04	1.43E-02	polyribonucleotide nucleotidyltransferase
PIOMA14_RS00895	PIOMA14_I_0182	atpB	77.23	-1.16	-2.23	6.12E-03	7.92E-02	hypothetical protein, F0F1 ATP synthase subunit A
PIOMA14_RS12725	PIOMA14_II_0499		101.21	-1.16	-2.23	1.73E-02	1.50E-01	type IA DNA topoisomerase
PIOMA14_RS03005	PIOMA14_I_0599		99.28	-1.15	-2.22	2.74E-02	1.97E-01	mannose-1-phosphate guanylyltransferase
PIOMA14_RS08095	PIOMA14_I_1602		42.70	-1.15	-2.21	1.69E-02	1.48E-01	TonB-dependent receptor
PIOMA14_RS00540	PIOMA14_I_0108		256.45	-1.14	-2.21	5.95E-02	3.05E-01	metallophosphoesterase family protein
PIOMA14_RS08815	PIOMA14_I_1743		79.09	-1.14	-2.21	1.03E-02	1.09E-01	TonB-dependent receptor
PIOMA14_RS01320	PIOMA14_I_0267	mtgA	51.95	-1.14	-2.20	9.21E-02	3.80E-01	monofunctional biosynthetic peptidoglycan transglycosylase
PIOMA14_RS10495	PIOMA14_II_0064	nqrE	106.78	-1.14	-2.20	4.88E-02	2.71E-01	NADH:ubiquinone reductase (Na(+)-transporting) subunit E
PIOMA14_RS03955	PIOMA14_I_0778		140.58	-1.13	-2.19	2.41E-02	1.85E-01	DUF4922 domain-containing protein
PIOMA14_RS03035	PIOMA14_I_0605		2263.17	-1.13	-2.18	3.11E-04	9.82E-03	TetR/AcrR family transcriptional regulator
PIOMA14_RS04445	PIOMA14_I_0871		87.23	-1.12	-2.18	9.59E-02	3.86E-01	DUF5606 domain-containing protein
PIOMA14_RS03925	PIOMA14_I_0773		128.40	-1.12	-2.18	3.58E-02	2.29E-01	DUF4494 domain-containing protein
PIOMA14_RS01960	PIOMA14_I_0388		103.63	-1.12	-2.18	3.31E-02	2.19E-01	methionyl-tRNA formyltransferase
PIOMA14_RS04000	PIOMA14_I_0786		23.53	-1.12	-2.17	7.28E-02	3.44E-01	hypothetical protein, DEAD/DEAH box helicase
PIOMA14_RS03065	PIOMA14_I_0611	gcvH	294.30	-1.11	-2.17	9.42E-03	1.03E-01	glycine cleavage system protein GcvH
PIOMA14_RS01325	PIOMA14_I_0268		534.04	-1.11	-2.16	3.05E-04	9.77E-03	UvrD-helicase domain-containing protein
PIOMA14_RS01285	PIOMA14_I_0260	pyrF	53.09	-1.11	-2.16	6.68E-02	3.27E-01	orotidine-5'-phosphate decarboxylase
PIOMA14_RS00220	PIOMA14_I_0046	hutH	237.74	-1.11	-2.15	2.27E-07	2.36E-05	imidazolonepropionase, histidine ammonia-lyase
PIOMA14_RS08000	PIOMA14_I_1583	sufC	46.36	-1.10	-2.15	5.66E-02	2.94E-01	Fe-S cluster assembly ATPase SufC
PIOMA14_RS04380	PIOMA14_I_0858		459.63	-1.10	-2.14	1.74E-04	5.96E-03	cation:proton antiporter
PIOMA14_RS11430	PIOMA14_II_0226		354.13	-1.10	-2.14	8.47E-02	3.72E-01	site-specific integrase
PIOMA14_RS07605	PIOMA14_I_1498		53.49	-1.09	-2.13	1.78E-02	1.53E-01	TrkH family potassium uptake protein
PIOMA14_RS03060	PIOMA14_I_0610	gcvPA	183.53	-1.09	-2.12	2.02E-02	1.66E-01	aminomethyl-transferring glycine dehydrogenase subunit GcvPA
PIOMA14_RS03355	PIOMA14_I_0665	folD	1360.04	-1.08	-2.11	8.93E-02	3.76E-01	bifunctional methylenetetrahydrofolate dehydrogenase/methenyltetrahydrofolate cyclohydrolase FolD
PIOMA14_RS02610	PIOMA14_I_0524	def	306.75	-1.08	-2.11	4.39E-02	2.57E-01	peptide deformylase
PIOMA14_RS12730	PIOMA14_II_0500		17.38	-1.07	-2.10	2.66E-02	1.94E-01	DUF2958 domain-containing protein
PIOMA14_RS10435	PIOMA14_II_0051		48.13	-1.07	-2.10	2.89E-02	2.05E-01	PKD domain-containing protein
PIOMA14_RS02605	PIOMA14_I_0523		260.95	-1.06	-2.08	4.12E-02	2.51E-01	hypothetical protein
PIOMA14_RS00910	PIOMA14_I_0185		290.81	-1.06	-2.08	8.64E-02	3.73E-01	F0F1 ATP synthase subunit delta
PIOMA14_RS04270	PIOMA14_I_0837		117.42	-1.05	-2.07	2.49E-02	1.87E-01	elongation factor G
PIOMA14_RS04525	PIOMA14_I_0887		209.00	-1.04	-2.06	8.09E-04	1.91E-02	acyl-CoA dehydrogenase family protein
PIOMA14_RS11635	PIOMA14_II_0278	traK_3	147.67	-1.04	-2.06	3.78E-03	5.87E-02	conjugative transposon protein TraK
PIOMA14_RS04030	PIOMA14_I_0791		84.10	-1.04	-2.06	9.89E-02	3.91E-01	phosphomannomutase/phosphoglucomutase
PIOMA14_RS08905	PIOMA14_I_1759		110.44	-1.04	-2.05	3.54E-02	2.29E-01	hypothetical protein
PIOMA14_RS04090	PIOMA14_I_0802		27.94	-1.04	-2.05	8.21E-02	3.64E-01	choice-of-anchor J domain-containing protein
PIOMA14_RS06585	PIOMA14_I_1292		117.18	-1.04	-2.05	2.58E-02	1.91E-01	DUF3413 domain-containing protein
PIOMA14_RS04890	PIOMA14_I_0962		30.86	-1.03	-2.04	3.11E-02	2.14E-01	glycosyl hydrolase family 25
PIOMA14_RS10410	PIOMA14_II_0046		197.66	-1.02	-2.03	7.06E-03	8.43E-02	ATP-dependent Clp protease ATP-binding subunit, hypothetical protein
PIOMA14_RS09285	PIOMA14_I_1831	era	505.56	-1.02	-2.02	4.28E-02	2.54E-01	GTPase Era
PIOMA14_RS03655	PIOMA14_I_0724		199.79	-1.01	-2.02	2.68E-02	1.94E-01	tRNA (N(6)-L-threonylcarbamoyladenosine(37)-C(2))-methylthiotransferase MtaB, glycosyltransferase family 2 protein
PIOMA14_RS06315	PIOMA14_I_1237		286.11	-1.00	-2.01	5.19E-03	7.16E-02	Omp28-related outer membrane protein
PIOMA14_RS03420	PIOMA14_I_0677		698.32	-1.00	-2.00	4.26E-03	6.26E-02	zinc-ribbon domain-containing protein

[1] Gene expression level
[2] Ratio of OxyR mut vs WT
[3] P value <0.01
[4] FDR - Fasle Discovery Rate

Supplementary Table S8. Genes upregulated in *P. intermedia* OMA14 OxyR mutant in iron deplete conditions (2 ≥ fold, P≤0.1)

locus tag	old locus tag	gene name	[1]Max group mean	[2]Log_2 fold change	[2]Fold change	[3]P-value	[4]FDR p-value	product (Genome (CDS))
PIOMA14_RS06730	PIOMA14_I_1319		24.97	6.38	83.34	3.27E-02	2.18E-01	thiazole biosynthesis protein
PIOMA14_RS00785	PIOMA14_I_0161		37.68	5.77	54.54	4.96E-02	2.73E-01	cupin domain-containing protein
PIOMA14_RS12085	PIOMA14_II_0372		29.19	5.76	54.06	5.34E-02	2.84E-01	phage virion morphogenesis protein, hypothetical protein
PIOMA14_RS01380	PIOMA14_I_0280		5.42	5.66	50.43	5.44E-02	2.86E-01	type IA DNA topoisomerase
PIOMA14_RS08780	PIOMA14_I_1737		14.59	5.66	50.43	5.44E-02	2.86E-01	phosphatase PAP2 family protein
PIOMA14_RS08455	PIOMA14_I_1672	cas1	10.32	5.40	42.20	6.69E-02	3.27E-01	type II CRISPR-associated endonuclease Cas1
PIOMA14_RS05175	PIOMA14_I_1021		15.14	5.40	42.20	6.69E-02	3.27E-01	AAA family ATPase, hypothetical protein
PIOMA14_RS01430	PIOMA14_I_0291		3.83	5.09	33.97	8.52E-02	3.72E-01	YWFCY domain-containing protein
PIOMA14_RS00695	PIOMA14_I_0140		14.52	4.90	29.85	9.79E-02	3.90E-01	DUF4494 domain-containing protein
PIOMA14_RS01220	PIOMA14_I_0248		22.34	4.90	29.85	9.79E-02	3.90E-01	hypothetical protein
PIOMA14_RS06150	PIOMA14_I_1205		336.33	4.19	18.22	5.55E-16	4.62E-13	hypothetical protein
PIOMA14_RS01580	PIOMA14_I_0320		15.87	4.11	17.25	3.47E-03	5.58E-02	PcfJ domain-containing protein
PIOMA14_RS12515	PIOMA14_II_0452		26.89	3.95	15.42	4.18E-04	1.22E-02	hypothetical protein
PIOMA14_RS13050	PIOMA14_II_0581		60.54	3.64	12.46	1.07E-02	1.10E-01	protease inhibitor I9 family protein
PIOMA14_RS12335	PIOMA14_II_0418		18.30	3.43	10.76	1.36E-02	1.30E-01	hypothetical protein
PIOMA14_RS07025	PIOMA14_I_1378		25.23	3.26	9.57	1.76E-03	3.60E-02	DUF3781 domain-containing protein
PIOMA14_RS05170	PIOMA14_I_1020		16.89	3.19	9.15	2.27E-02	1.78E-01	hypothetical protein
PIOMA14_RS07795	PIOMA14_I_1536		22.99	3.19	9.13	1.94E-02	1.62E-01	DUF4280 domain-containing protein, hypothetical protein
PIOMA14_RS06430	PIOMA14_I_1260		7.94	3.12	8.67	5.59E-03	7.46E-02	TonB-dependent receptor
PIOMA14_RS08150	PIOMA14_I_1614		96.19	3.10	8.59	1.98E-02	1.64E-01	hypothetical protein
PIOMA14_RS08145			40.86	3.05	8.28	4.78E-09	8.52E-07	leucine-rich repeat protein
PIOMA14_RS11370	PIOMA14_II_0217		331.39	2.94	7.69	8.39E-05	3.27E-03	hypothetical protein
PIOMA14_RS08485	PIOMA14_I_1678		7.62	2.87	7.32	1.31E-02	1.27E-01	hypothetical protein
PIOMA14_RS09035	PIOMA14_I_1785		77.67	2.86	7.27	3.03E-05	1.48E-03	sigma-70 family RNA polymerase sigma factor, hypothetical protein
PIOMA14_RS14035	PIOMA14_II_0772		32.69	2.78	6.86	1.11E-02	1.13E-01	DUF1320 family protein
PIOMA14_RS08380	PIOMA14_I_1656		21.40	2.75	6.74	5.36E-02	2.84E-01	hypothetical protein
PIOMA14_RS01160	PIOMA14_I_0235		6.16	2.74	6.70	4.82E-02	2.71E-01	site-specific integrase
PIOMA14_RS11195	PIOMA14_II_0186		10.03	2.74	6.70	4.82E-02	2.71E-01	hypothetical protein
PIOMA14_RS05110	PIOMA14_I_1008		5.80	2.74	6.70	4.82E-02	2.71E-01	DUF935 family protein
PIOMA14_RS08675	PIOMA14_I_1717		12.09	2.74	6.70	4.82E-02	2.71E-01	thymidylate synthase
PIOMA14_RS05565	PIOMA14_I_1098		18.24	2.68	6.42	9.83E-03	1.05E-01	Type 1 glutamine amidotransferase-like domain-containing protein
PIOMA14_RS13225	PIOMA14_II_0620		1463.27	2.64	6.21	7.34E-05	3.03E-03	hypothetical protein, sigma-70 family RNA polymerase sigma factor
PIOMA14_RS00670	PIOMA14_I_0135		28.31	2.58	5.97	1.60E-02	1.43E-01	DUF1320 family protein
PIOMA14_RS07120	PIOMA14_I_1395		39.59	2.57	5.95	1.95E-02	1.62E-01	heavy metal-binding domain-containing protein
PIOMA14_RS00025	PIOMA14_I_0005		12.42	2.56	5.89	6.80E-02	3.27E-01	hypothetical protein
PIOMA14_RS14435	PIOMA14_II_0762		21.71	2.56	5.89	6.80E-02	3.27E-01	hypothetical protein
PIOMA14_RS00645	PIOMA14_I_0130		14.56	2.56	5.89	6.80E-02	3.27E-01	N-acetylmuramoyl-L-alanine amidase
PIOMA14_RS00735	PIOMA14_I_0148		11.03	2.56	5.89	6.80E-02	3.27E-01	ATP-dependent serine protease
PIOMA14_RS08420	PIOMA14_I_1664		7.01	2.56	5.89	6.80E-02	3.27E-01	HAD-IB family phosphatase
PIOMA14_RS08505	PIOMA14_I_1682		10.82	2.56	5.89	6.80E-02	3.27E-01	hypothetical protein
PIOMA14_RS09040	PIOMA14_I_1786		105.40	2.56	5.88	5.13E-06	3.50E-04	hypothetical protein
PIOMA14_RS14160			117.67	2.51	5.68	7.05E-03	8.43E-02	hypothetical protein
PIOMA14_RS11400	PIOMA14_II_0221	interpain A	240.16	2.50	5.65	1.14E-06	8.89E-05	C10 family peptidase
PIOMA14_RS11140	PIOMA14_II_0174		9.49	2.46	5.52	1.94E-02	1.62E-01	restriction endonuclease subunit S
PIOMA14_RS00625	PIOMA14_I_0126		24.07	2.46	5.52	1.90E-02	1.60E-01	hypothetical protein, BACON domain-containing protein
PIOMA14_RS07005	PIOMA14_I_1373		20.78	2.43	5.38	2.18E-02	1.74E-01	class I SAM-dependent methyltransferase
PIOMA14_RS06225	PIOMA14_I_1222		23.00	2.41	5.30	6.74E-03	8.38E-02	hypothetical protein
PIOMA14_RS02095	PIOMA14_I_0415		41.90	2.40	5.27	2.83E-02	2.01E-01	hypothetical protein
PIOMA14_RS02895	PIOMA14_I_0577		544.02	2.38	5.22	7.86E-06	4.90E-04	Crp/Fnr family transcriptional regulator
PIOMA14_RS13220	PIOMA14_II_0619		1269.33	2.36	5.14	7.53E-04	1.83E-02	hypothetical protein, sigma-70 family RNA polymerase sigma factor
PIOMA14_RS02255	PIOMA14_I_0447		78.25	2.35	5.10	7.07E-02	3.38E-01	helix-turn-helix transcriptional regulator
PIOMA14_RS13060	PIOMA14_II_0583		5.19	2.34	5.08	9.83E-02	3.90E-01	ISL3 family transposase
PIOMA14_RS10020	PIOMA14_I_1967		11.86	2.34	5.08	9.83E-02	3.90E-01	S-ribosylhomocysteine lyase
PIOMA14_RS00865	PIOMA14_I_0177		5.84	2.34	5.08	9.83E-02	3.90E-01	oligosaccharide flippase family protein, glycosyltransferase family 2 protein
PIOMA14_RS11150	PIOMA14_II_0176		11.34	2.34	5.07	1.00E-01	3.94E-01	restriction endonuclease subunit S
PIOMA14_RS09145	PIOMA14_I_1803		10.31	2.34	5.07	2.68E-02	1.94E-01	nucleotidyl transferase AbiEii/AbiGii toxin family protein
PIOMA14_RS07360	PIOMA14_I_1445		8.44	2.34	5.07	2.68E-02	1.94E-01	hypothetical protein
PIOMA14_RS11120	PIOMA14_II_0152		16.33	2.34	5.07	2.68E-02	1.94E-01	hypothetical protein
PIOMA14_RS13990	PIOMA14_II_0763		22.09	2.34	5.07	2.68E-02	1.94E-01	hypothetical protein, BACON domain-containing protein
PIOMA14_RS03100	PIOMA14_I_0617		47.72	2.34	5.06	3.79E-03	5.87E-02	histidine phosphatase family protein
PIOMA14_RS11395	PIOMA14_II_0220		119.93	2.33	5.04	8.80E-06	5.36E-04	choice-of-anchor J domain-containing protein
PIOMA14_RS06135	PIOMA14_I_1203		21.63	2.32	5.01	1.24E-06	9.36E-05	choice-of-anchor J domain-containing protein
PIOMA14_RS02525	PIOMA14_I_0508		11.77	2.25	4.75	3.52E-02	2.29E-01	site-specific integrase
PIOMA14_RS05270	PIOMA14_I_1039		22.72	2.23	4.68	5.10E-03	7.07E-02	HlyD family efflux transporter periplasmic adaptor subunit
PIOMA14_RS07420	PIOMA14_I_1458		13.10	2.21	4.62	3.84E-02	2.37E-01	hypothetical protein
PIOMA14_RS06880	PIOMA14_I_1348		9.90	2.21	4.62	3.84E-02	2.37E-01	phosphatase PAP2 family protein
PIOMA14_RS12480	PIOMA14_II_0445		22.03	2.20	4.59	6.61E-03	8.29E-02	peptidase
PIOMA14_RS12835	PIOMA14_II_0522		10.73	2.20	4.59	7.16E-03	8.43E-02	hypothetical protein
PIOMA14_RS08480	PIOMA14_I_1677		22.96	2.15	4.44	1.80E-02	1.54E-01	hypothetical protein
PIOMA14_RS12845	PIOMA14_II_0529		19.38	2.14	4.40	7.16E-04	1.75E-02	hypothetical protein
PIOMA14_RS04760	PIOMA14_I_0937		43.71	2.14	4.40	7.63E-04	1.83E-02	formylglycine-generating enzyme family protein
PIOMA14_RS08555	PIOMA14_I_1693		48.41	2.10	4.29	1.07E-02	1.10E-01	phage virion morphogenesis protein, hypothetical protein
PIOMA14_RS08595	PIOMA14_I_1701		20.00	2.10	4.29	1.31E-02	1.27E-01	hypothetical protein

PIOMA14_RS05275			95.29	2.08	4.22	1.60E-04	5.62E-03	TolC family protein
PIOMA14_RS01680	PIOMA14_I_0339		28.71	2.07	4.20	5.39E-04	1.40E-02	TonB-dependent receptor family protein, tellurium resistance protein TerC
PIOMA14_RS13210	PIOMA14_II_0617		590.44	2.06	4.18	4.74E-06	3.38E-04	outer membrane beta-barrel protein
PIOMA14_RS02260			33.54	2.06	4.17	5.57E-02	2.92E-01	helicase
PIOMA14_RS02800	PIOMA14_I_0558		10.38	2.06	4.17	5.66E-02	2.94E-01	aldo/keto reductase
PIOMA14_RS13075	PIOMA14_II_0588		10.27	2.05	4.13	2.26E-02	1.78E-01	IS4 family transposase
PIOMA14_RS00055	PIOMA14_I_0011		26.21	2.04	4.11	2.43E-02	1.86E-01	carboxypeptidase-like regulatory domain-containing protein, radical SAM protein
PIOMA14_RS13010			8.96	2.04	4.11	2.40E-02	1.85E-01	DNA methylase
PIOMA14_RS11790	PIOMA14_II_0310		22.65	2.03	4.09	7.64E-03	8.72E-02	hypothetical protein, relaxase/mobilization nuclease domain-containing protein
PIOMA14_RS08730	PIOMA14_I_1728		37.76	2.02	4.06	1.51E-02	1.38E-01	GLPGLI family protein

[1] Gene expression level
[2] Ratio of OxyR mut vs WT
[3] P value < 0.01
[4] FDR - False Discovery Rate

Supplementary Figures:

Supplementary Figure 1. Comparison of the genomic loci encoding OxyR in *Prevotella* species.
The location and directions of genes (arrows) in oxyR loci of *Prevotella intermedia* strains (A) and *Prevotella* species (B). Color coding represents % homology based on amino acid sequences of genes calculated by GenomeMatcher [Ref. 1].

Supplementary Figure 2. Distribution of SOD and glutathione peroxidase genes within the genus *Prevotella*.
The presence of SOD gene (open triangles) and glutathione peroxidase genes (black triangles: *gpx1*, PIOMA14_I_0630 type, gray triangles: *gpx2*, PIOMA14_I_1325 type) are shown in the 16S-rRNA gene sequence-based phylogenetic tree of the genus *Prevotella* (adapted from Sakamoto *et al.* [Ref. 2]).

References:

1. Ohtsubo, Y., Ikeda-Ohtsubo, W., Nagata, Y., & Tsuda, M. GenomeMather: A graphical user interface for DNA sequence comparison. *BMC Bioinformatics* **9**:376 (2008)

2. Sakamoto, M., Suzuki, N., & Okamoto, M. *Prevoella aurantiaca* sp. Nov., isolated from the human oral cavity. *Int. J. Syst. Evol. Microbiol.* **60**, 500-503 (2010).
I add legends for supplement figures.

Sup. Fig. 1a

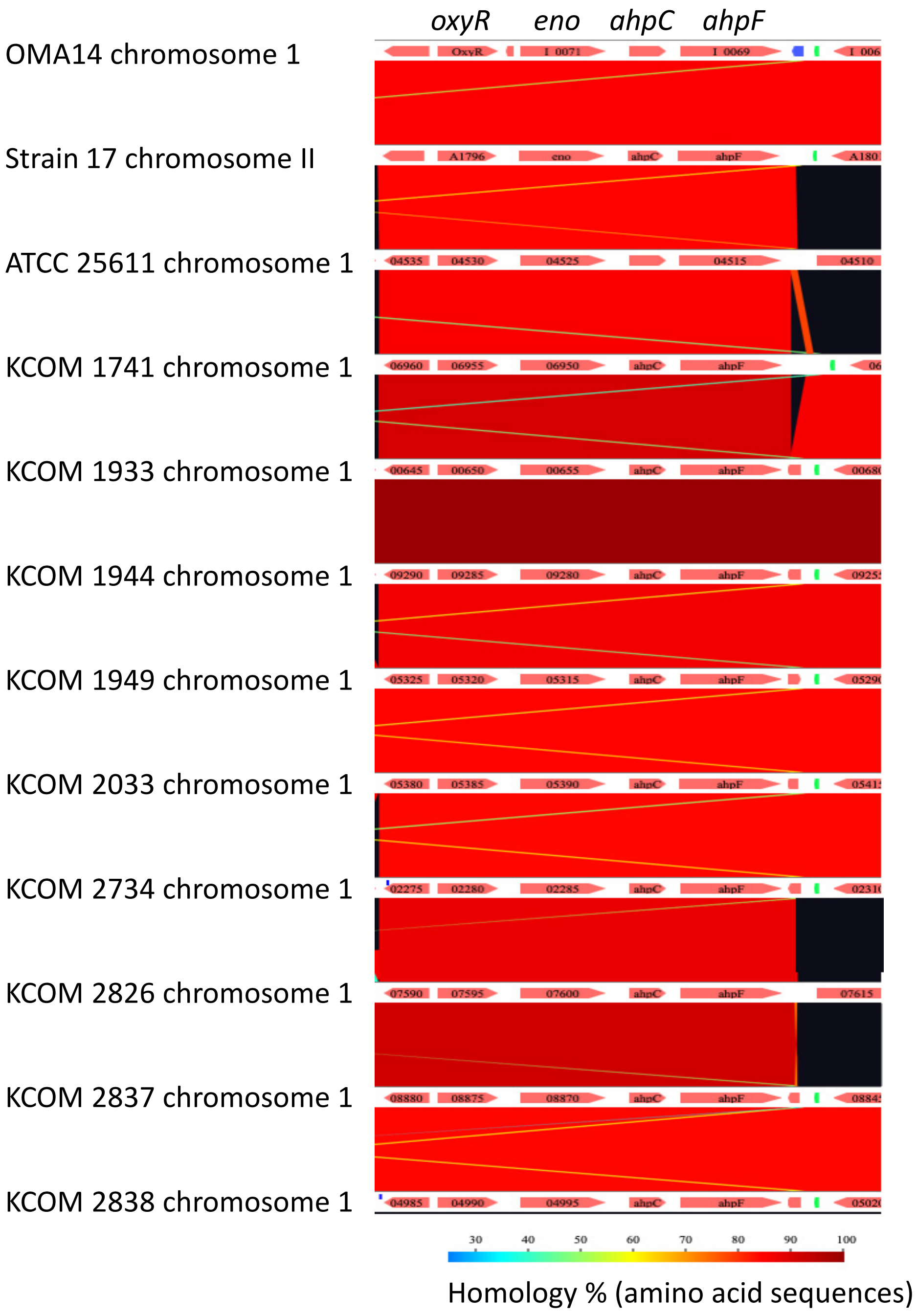

oxyR
eno
ahpC
ahpF
OMA14 chromosome 1
Strain 17 chromosome II
ATCC 25611 chromosome 1
KCOM 1741 chromosome 1
KCOM 1933 chromosome 1
KCOM 1944 chromosome 1
KCOM 1949 chromosome 1
KCOM 2033 chromosome 1
KCOM 2734 chromosome 1
KCOM 2826 chromosome 1
KCOM 2837 chromosome 1
KCOM 2838 chromosome 1
30
40
50
60
70
80
90
100
Homology % (amino acid sequences)

Sup. Fig. 1b

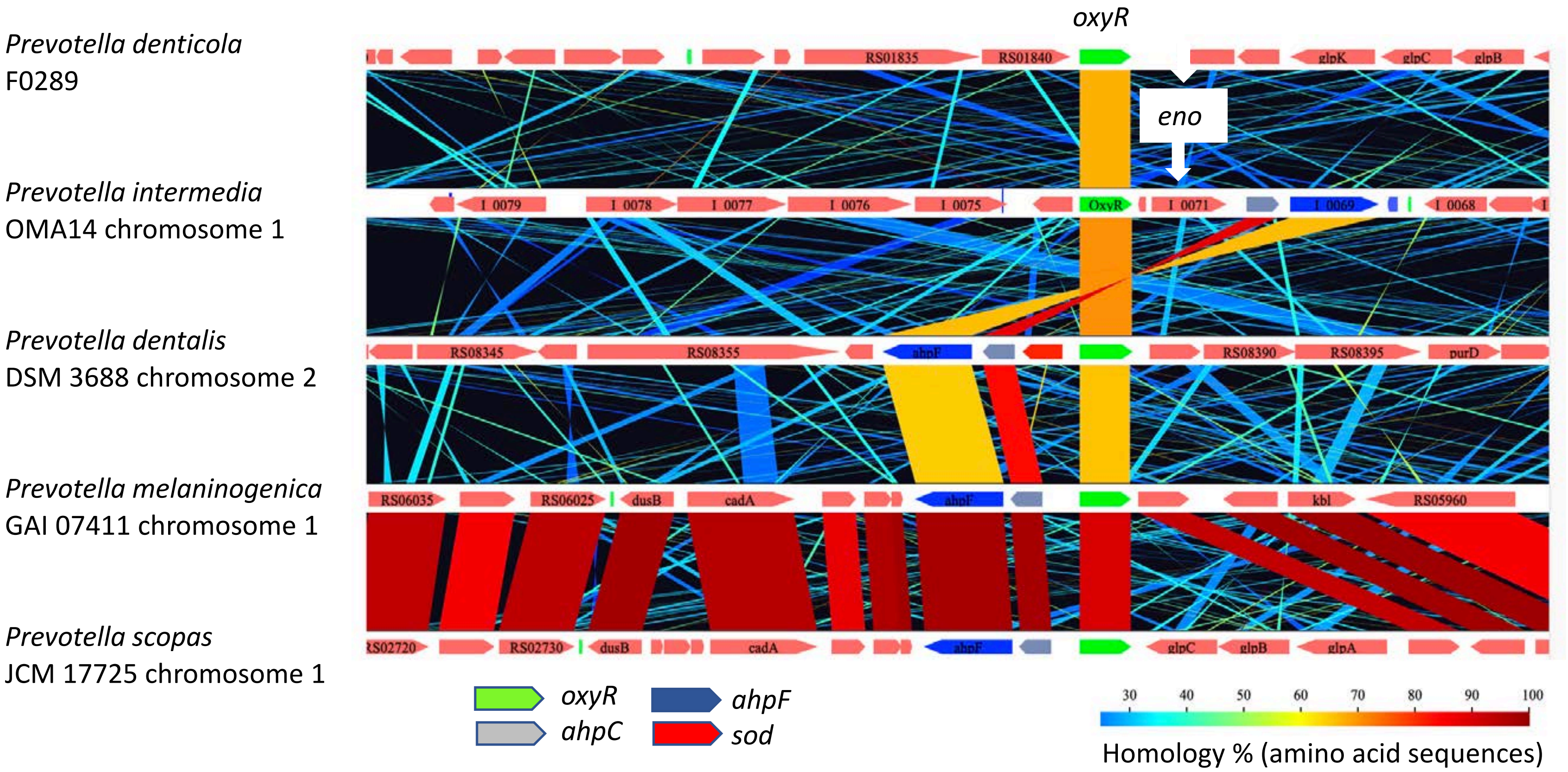

oxyR
Prevotella denticola F0289
eno
Prevotella intermedia OMA14 chromosome 1
Prevotella dentalis DSM 3688 chromosome 2
Prevotella melaninogenica GAI 07411 chromosome 1
Prevotella scopas JCM 17725 chromosome 1
oxyR
ahpC
ahpF
sod
30 40 50 60 70 80 90 100
Homology % (amino acid sequences)

Sup. Fig. 2

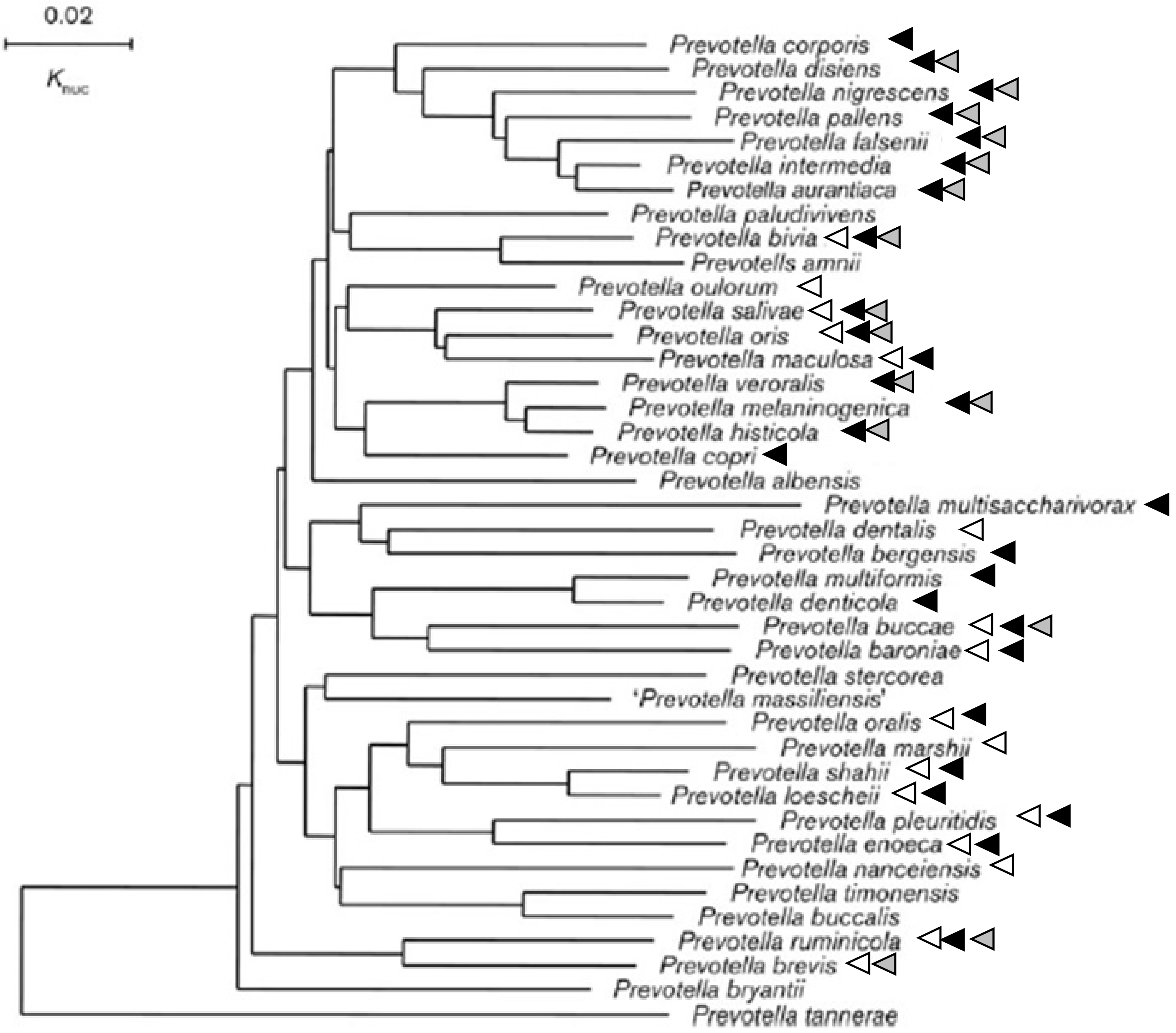

0.02
K_{nuc}
Prevotella corporis
Prevotella disiens
Prevotella nigrescens
Prevotella pallens
Prevotella falsenii
Prevotella intermedia
Prevotella aurantiaca
Prevotella paludivivens
Prevotella bivia
Prevotells amnii
Prevotella oulorum
Prevotella salivae
Prevotella oris
Prevotella maculosa
Prevotella veroralis
Prevotella melaninogenica
Prevotella histicola
Prevotella copri
Prevotella albensis
Prevotella multisaccharivorax
Prevotella dentalis
Prevotella bergensis
Prevotella multiformis
Prevotella denticola
Prevotella buccae
Prevotella baroniae
Prevotella stercorea
'Prevotella massiliensis'
Prevotella oralis
Prevotella marshii
Prevotella shahii
Prevotella loescheii
Prevotella pleuritidis
Prevotella enoeca
Prevotella nanceiensis
Prevotella timonensis
Prevotella buccalis
Prevotella ruminicola
Prevotella brevis
Prevotella bryantii
Prevotella tannerae
